# Multifunctional protective strategies for wooden cultural heritage: antimicrobial efficacy of polyacrylic resins, siloxane coupling agents, and silver nanoparticles

**DOI:** 10.3389/fmicb.2025.1642335

**Published:** 2025-08-22

**Authors:** Andreea Ștefania Dumbravă, Viorica Maria Corbu, Ioana Cristina Marinaș, Radu Pericleanu, Liliana Marinescu, Ludmila Motelica, Doina Roxana Trusca, Nicoleta Ianovici, Tatiana Eugenia Șesan, Irina Gheorghe-Barbu, Denisa Ficai, Ovidiu Cristian Oprea, Anton Ficai, Mariana Carmen Chifiriuc

**Affiliations:** ^1^Department of Microbiology and Botany, Faculty of Biology, University of Bucharest, Bucharest, Romania; ^2^Department of Technological Irradiation (IRASM), Horia Hulubei National Institute of Physics and Nuclear Engineering–IFIN-HH, Magurele, Romania; ^3^Research Institute of the University of Bucharest, University of Bucharest, Bucharest, Romania; ^4^Department of Genetics, Faculty of Biology, University of Bucharest, Bucharest, Romania; ^5^Faculty of Chemical Engineering and Biotechnologies, National University of Science and Technology Politehnica Bucharest, Bucharest, Romania; ^6^Research Center for Advanced Materials, Products and Processes, National University of Science and Technology Politehnica Bucharest, Bucharest, Romania; ^7^Academy of Romanian Scientist, Bucharest, Romania; ^8^National Center for Micro and Nanomaterials, National University of Science and Technology Politehnica Bucharest, Bucharest, Romania; ^9^Department of Biology, Faculty of Chemistry, Biology, Geography, Environmental Biology and Biomonitoring Research Center, West University of Timisoara, Timisoara, Romania; ^10^Romanian Academy of Agricultural and Forestry Sciences, Bucharest, Romania

**Keywords:** wood heritage objects, siloxane-based coupling agent, silver nanoparticles, polyacrylic resins, antimicrobial, anti-biofilm, anti-metabolic

## Abstract

**Introduction:**

This study evaluates two innovative protective treatments for wooden cultural heritage objects vulnerable to biodeterioration. The first involves polyacrylic resin solutions embedded with silver nanoparticles (AgNPs), while the second uses the siloxane-based coupling agent 3-mercaptopropyltrimethoxysilane (3-MPTMS) to enhance AgNP adhesion to wood surfaces.

**Methods:**

Antimicrobial, anti-biofilm, and anti-metabolic activities were assessed using both qualitative and quantitative assays against biodeteriogenic strains (*Penicillium chrysogenum, Bacillus cereus*, and *Bacillus megaterium*). Additional analyses included extracellular nitric oxide (NO) production, phytotoxicity testing on *Allium cepa*, and correlations with biochemical parameters.

**Results:**

Polyacrylic resins incorporating AgNPs exhibited significant antimicrobial properties, with stronger effects against bacteria. Treatments combining AgNPs with MPTMS or acrylic resins demonstrated enhanced inhibitory effect on microbial viability, adhesion, and degradative enzymes secretion. Sub-inhibitory concentrations of resin solutions modulated the extracellular NO levels, correlated with metabolic stress responses. Notably, ecotoxicity testing confirmed minimal phytotoxic impact, supporting the safety of these materials for cultural heritage applications.

**Conclusion:**

The findings support the use of AgNP-enhanced polyacrylic resins and silanized coatings as effective, non-destructive conservation strategies for wooden heritage artifacts, offering durable protection against microbial deterioration while maintaining environmental safety.

## 1 Introduction

Preserving wood heritage objects presents a formidable challenge for conservators and researchers, given their high susceptibility to biodeterioration, a process predominantly driven by microbial activity. As an organic material, wood is particularly prone to colonization by fungi, bacteria, and other microorganisms, leading to significant structural damage, discoloration, and a gradual loss of cultural and historical value (Cappitelli et al., 2020). Microbial deterioration compromises the physical integrity of the artifacts and erodes the cultural narratives they represent. The microbial community involved in wood artifacts degradation comprises a variety of microbial species, each of them presenting specific degradation mechanisms (Schrader et al., 2025). For instance, species such as *Bjerkandera adusta*, *Ganoderma lucidum*, *Irpex lacteus*, *Phanerochaete chrysosporium*, *Pleurotus ostreatus*, and *Trametes versicolor* classified as white rot fungi compromise the structural integrity of wooden artifacts through the secretion of lignocellulolytic enzymes, including endoglucanases, cellobiohydrolases, and β-glucosidases. In contrast, brown rot fungi such as *Antrodia vaillantii*, *Coniophora puteana*, *Gloeophyllum trabeum*, and *Serpula lacrymans* produce non-enzymatic compounds such as oxalic acid which diffuses in the plant cell wall and then generates reactive oxygen species such as hydroxyl radicals that depolymerize cellulose and hemicellulose (Martínez et al., 2005; Eastwood et al., 2011; Alfieri et al., 2021; Schrader et al., 2025). In addition to fungi, bacteria contribute notably to the decay of wooden artifacts, especially in environments with limited oxygen availability—such as waterlogged, buried, or highly humid conditions—where their metabolic activity becomes more pronounced. Depending on their mechanisms of wood tissue invasion, biodeteriorating bacteria are classified into several functional groups, including cavitation bacteria, tunneling bacteria, and erosion bacteria—each exhibiting distinct metabolic adaptations that enable their survival under specific environmental conditions (Chu et al., 2025; Wang et al., 2025). Regardless of their classification, the growing demand for effective conservation measures underscores the urgent need for innovative strategies that can provide long-lasting protection against microbial degradation while preserving the structural and esthetic integrity of the wood (Cirone et al., 2023; Pournou, 2020).

Polyacrylic resins have been widely used in the conservation of wooden artifacts due to their adhesive properties and ability to form protective coatings as well as stability (Kyei et al., 2022). Overall, polyacrylic resins are used in the treatment of the wood heritage because of its capability to bind to the wood structure and even to consolidate the fragile wood structure, to protect against moisture, UV or even to assure a higher scratch-resistance (especially as composite with silica, glass or wood flour) and these effects persist for long time. They are reversible and insoluble in water but dissolve readily in organic solvents, which are commonly employed in conserving wooden artworks. While highly resistant to extremes temperature, acids, and alkali, acrylic resins can undergo degradation over time from factors such as microbial activity, UV radiation, and mechanical wear, leading to alterations in their physical and mechanical characteristics (Cataldi et al., 2014; Cataldi et al., 2015). Nonetheless, the use of these treatments alone frequently fails to provide comprehensive protection against microbial colonization and the ensuing biodeterioration processes (Campana et al., 2022; Ion et al., 2018). Ongoing researches are now underway to pass from the traditionally petrochemical based acrylic resins to bio-based feedstock (Wen et al., 2023).

Recent advancements in improving the protective performance of polyacrylic resins have emphasized the integration of antimicrobial agents, including nanoparticles such as silver (AgNPs), titanium dioxide (TiO₂), and zinc oxide (ZnO) (Arya et al., 2019; De Caro et al., 2024). For instance, TiO₂ and SiO₂ nanoparticles integrated into acrylic resins significantly suppressed *Streptococcus mutans* and *Lactobacillus acidophilus*, obtaining reductions of 98–99% in microbial viability under UV exposure (Sodagar et al., 2016). Similarly, ZnO NPs exhibit significant antimicrobial properties, effectively inhibiting bacterial and fungal colonization when incorporated into thin-film coatings or applied to heritage materials like paper and stone (Cinteză and Tănase, 2021). Recent investigations have shown that the incorporation of AgNPs into acrylic or silane-modified resin systems considerably improves antibacterial protection on cultural heritage substrates. In a 2024 study, AgNPs were biosynthesised *in situ* during the synthesis of an acrylic polymer coating (by plant extract and AgNO₃ mixing). The resulting nanocomposite decreased microbial load on stone heritage models by 2–4 log units compared to acrylic alone (Lak et al., 2024). Furthermore, a treatment combining a polyvinyl chloride-abietic acid (PVC-AA) copolymer with AgNPs was developed to improve the characteristics of pine wood, with a focus on dimensional stability, water repellence, and decay resistance. The results showed that while the impregnation procedure did not improve the wood’s physical qualities, the PVC-AA-AgNPs treatment did greatly increase resistance to *T. versicolor* and *C. puteana*, demonstrating its potential as a protective treatment for wooden historical objects (Can and Hazer, 2022).

AgNPs are recognized for their high antimicrobial activity and broad-spectrum efficacy, being effective against bacteria, fungi, and viruses. These features make AgNPs an ideal additive for conservation materials, including resins, specifically designed to protect wood from microbial colonization and subsequent damage and deterioration (Baglioni et al., 2021). The antimicrobial properties of AgNPs are attributed to their ability to release silver ions, which can interact with microbial cell membranes, proteins, and DNA, leading to cell damage and death (Rai et al., 2009). Despite their efficacy, the integration of AgNPs into conservation materials presents significant challenges. One primary concern is represented by NPs cytotoxicity, which could adversely impact both the artifacts and the surrounding environment. Consequently, their use in conservation requires careful assessment and rigorous evaluation to balance their protective benefits against any possible risks (Silva et al., 2017). One significant concern is the potential for phytotoxic effects, which could arise from the release of silver ions or other chemical interactions involving the siloxane-based coupling agents. Phytotoxicity could lead to unintended damage to the wood or surrounding organic materials, compromising the long-term safety and integrity of the conserved artifacts. Therefore, a comprehensive evaluation of these materials must include not only the assessment of their antimicrobial efficacy but also of their potential impact on the artifacts and the environment (Ameen et al., 2021). Plant-based (green synthesis) AgNPs (PB-AgNPs) offers a biocompatible and environmentally sustainable alternative to conventional chemical methods. This process utilizes phytochemicals such as polyphenols, flavonoids, and terpenoids as natural reducing and capping agents, enabling NP formation under mild conditions while significantly reducing ecological and human health risks (Iravani et al., 2014). Biologically produced AgNPs are often regarded as eco-friendly alternatives because they minimize the use of hazardous chemicals. However, they can still present ecotoxicological concerns, including alterations to soil microbial communities, accumulation in plant tissues, and disruption of key ecological processes such as nitrogen cycling and organic matter breakdown (Colman et al., 2014; Ottoni et al., 2020). Among the biological agents used for green synthesis, are particularly advantageous due to their rapid growth, ability to secrete bioactive enzymes, and natural resistance to heavy metals. Various fungal species, such as *Fusarium*, *Aspergillus*, *Trichoderma*, *Cladosporium*, *Ganoderma*, and the wood-degrading *Gloeophyllum striatum* have been effectively utilized in the biosynthesis of AgNPs with antimicrobial properties (Cui et al., 2022; Wrońska et al., 2023; Al-Zaban et al., 2021; Osorio-Echavarría et al., 2021; Santos et al., 2021; Zawadzka et al., 2021; Tończyk et al., 2025).

In terms of biological safety, NPs exhibit dose-dependent and cell-type-specific cytotoxicity. Cytotoxicity mechanisms often involve oxidative stress, DNA damage, and cell cycle arrest through interactions with proteins and nucleic acids (Ribeiro et al., 2018). Factors such as particle size, crystallinity, surface functionalization, and aging-related silver ion release further modulate toxicity (Wang et al., 2014; Kittler et al., 2010; Siddiqi et al., 2018). AgNPs synthesized using *Pseudomonas putida* showed no cytotoxicity on Hep-2 cells at concentrations up to 25 μg/mL, but caused 70% cell death at 100 μg/mL (Gopinath et al., 2017). Similarly, Khorrami et al. (2018) found that biosynthesized AgNPs induced 70% cell death in MCF-7 breast cancer cells at 60 μg/mL, while exhibiting much lower toxicity toward noncancerous L-929 cells. The lower toxicity compared to other commercial NPs could be possibly due to better encapsulation and biocompatible surface coatings (Khan et al., 2020). The minimal toxicity to normal cells at low doses reinforces the potential of green AgNPs for selective anticancer applications (Ghabban et al., 2022).

Siloxane-based coupling agents are organosilane compounds (OS) that exhibit very good properties like fire retardancy, resistance to microbial degradation, and increased weathering stability (Liu et al., 2023). Wood substrates treated with OS demonstrate improved functional performance, since these agents can enhance the surface hydrophobicity, thereby reducing moisture absorption—a critical factor in microbial growth and biodeterioration (Aziz et al., 2021; Alfieri et al., 2021). OS are derived from silane (SiH₄) by substituting one or more hydrogen atoms with organic or functional groups, yielding the general formula RₙSiH₄₋ₙ (e.g., trymethylsilane, tetravinylsilane, trimethylsilylamine, chrolosilane, trimethoxysilane) (Liu et al., 2023; Broda et al., 2019; Panov and Terziev, 2009). These R groups—such as alkyl, amino, sulfhydryl, methoxy, glycidyloxy, or vinyl—provide compatibility with organic materials, making OS compounds effective coupling agents between organic and inorganic phases. Given their bi-functional molecular structure, upon hydrolysis, one end of the silane molecule reacts with inorganic substrates or hydroxyl-rich wood surfaces, while the other end may engage in covalent bonding with polymer matrices. This amphiphilic nature enhances interfacial compatibility between nanoparticles and polymeric phases (Jamil et al., 2018). Recent studies have highlighted the synergistic potential of OS compounds, especially when combined with nanoparticles or other bioactive agents, in enhancing wood durability. OS-treated wood is increasingly utilized across diverse sectors, including structural engineering, architectural finishing, furniture manufacturing, electronic components, and aerospace materials (Liu et al., 2023).

By combining AgNPs with silanization agents, it is possible to create a dual-functioning resin that not only inhibits microbial colonization but also strengthens the physical barrier against environmental factors that contribute to wood degradation (Ferreira et al., 2023; Nayaki et al., 2023). Similarly, OS treatments might enhance the biological resistance of thermally modified wood. Although thermal treatment reduces moisture content and increases initial resistance to biological agents, this effect diminishes over time. The addition of silane treatments post-thermal modification was found to extend and reinforce the wood’s resistance to microbial attack (Kamperidou, 2019). Oscillating techniques are used to improve OS treated wood durability, water resistance and to assure biological protection.

In this context, the present study aims to evaluate the efficacy of two distinct protective formulations for wood heritage conservation. The first formulation is based on polyacrylic resins incorporating AgNPs, while the second involves surface modification using the siloxane-based coupling agents 3-mercaptopropyltrimethoxysilane (3-MPTMS), to facilitate enhanced binding of AgNPs. Both formulations are intended to protect wooden heritage objects from biodeterioration. The study focuses on investigating their antimicrobial, anti-biofilm, and anti-metabolic activities and their correlation with microbial and biochemical parameters, using treated wood model systems. Additionally, the formulations are evaluated for their preservation efficacy and potential phytotoxicity, with the overarching goal of assessing their suitability as integrated solutions for extending the longevity of wooden artifacts susceptible to microbial degradation.

## 2 Materials and methods

### 2.1 Synthesis of AgNPs

Synthesis of AgNPs using *Classical method, at room temperature* (AgNPc), *Turkevich method* (by reduction in the presence of appropriate agents – AgNPt), and *hydrothermal method* (AgNPsol) was described in our previous work (Corbu et al., 2023).

Silver nitrate (AgNO_3_) was purchased from Sigma Aldrich, polyvinylpyrrolidone (PVP), from Roth, and trisodium citrate (Na_3_C_6_H_5_O_7_) from Alfa Aesar. All reagents and solvents were used without further purification.

#### 2.1.1 Synthesis of AgNPs by the classical method, at room temperature (AgNPc)

A volume of 500 mL of 0.0001 M AgNO_3_ solution was introduced into a Berzelius beaker, then 30 mL of 0.02999 M trisodium citrate was added. The solution thus obtained was left under continuous agitation (600–700 rpm) for 12 min, at room temperature. After 12 min, 30 mL of 0.00007 M PVP was added. The solution was stirred for 1 min (200 rpm), after which 3 or 5 mL of 0.1 M sodium borohydride solution was added. The final mixture was stirred for 5–7 min, after which 1.2 mL of 30% hydrogen peroxide was added. After adding H_2_O_2_, the obtained solution was stirred until a blue or emerald blue color appeared. The concentration of the obtained NPs was 10 ppm.

#### 2.1.2 Synthesis of AgNPs by the Turkevich method (AgNPt)

An amount of 0.01 g AgNO_3_ was dissolved in 500 mL H_2_O. The obtained solution was kept at 70–75° C under magnetic stirring for 75 min. To this solution was added dropwise a solution containing 0.30 g sodium salt of citric acid as reducing agent. When the solution became slightly yellowish, 10 mL of PVP were added. The concentration of the obtained NPs was 100 ppm.

#### 2.1.3 Synthesis of AgNPs by the solvothermal method (AgNPsol)

An amount of 0.888 g of PVP K 30 was added to 80 mL of PEG 400. The mixture was stirred and heated to 80° C until the obtained solution became transparent. When the temperature reached 80° C, 2 mL of AgNO_3_ with a concentration of 0.5 M was quickly added to the mixture, under stirring. A dark yellow color was observed, and the mixing continued at 80° C until the appearance of a dark brown color. The mixture was poured into a Teflon vat, and the temperature increased to 220° C while maintaining a constant pressure of 1 bar for 2 h. The reddish-brown mixture was cooled and then removed from the vat. The concentration of the obtained NPs was 1,000 ppm.

### 2.2 Treatment of wooden materials

#### 2.2.1 Treatment with *3-MPTMS* of wooden materials

In the case of treatment with 3-MPTMS (silane with –SH side group), the wood samples were introduced in 35 mL silanization solution (3-MPTMS), in a Berzelius flask and left under continuous stirring (600–700 rpm) 1 h, at room temperature. Subsequently, the wood samples were put in contact with the solution with Ag solution of concentration 100 ppm (Ag100) for 1 h under continuous stirring (600–700 rpm). In the final the wooden samples were removed from AgNPs solution and dried at room temperature.

#### 2.2.2 Treatment with based acrylic resin of wooden materials

To be silanized, the beech wood materials were washed and dried at 115° C for 2 h. After washing and drying, the surfaces of standardized wood samples (1*1 cm^2^) were impregnated using a painting brush with a commercial water-based acrylic resin, Adicril ARD-795, containing 78% acrylic binder from Bear Química, with or without AgNPs.

The samples were processed using a commercial water-based acrylic emulsion, Adicril ARD-795, containing 78% acrylic binder from Bear Química. The obtained emulsion was then mixed for 20 min using a Dispermat VMA mixer equipped with a 60 mm diameter blade, operating at 1,000 rpm, with AgNPs at a concentration of 10 ppm and 1,000 ppm. Four samples were obtained according to Supplementary Table S1. Both AgNPc samples, designated as AgNPc 3 mL (D1) and AgNPc 5 mL (D1), were synthesized through the reduction of silver nitrate using sodium borohydride (NaBH₄) and sodium citrate. The primary difference between these samples lies in the varying sodium borohydride content used during the synthesis of AgNP (Marinescu et al., 2022).

All samples contain acrylic binder and three of them contain different percentage of AgNPs characterized by variation of concentration as indicated in Supplementary Table S1.

#### 2.2.3 Characterization of wooden surfaces and treated materials

Morphological and structural characterization of model surfaces and treated materials were achieved using specific physico-chemical methods: IR microscopy, Dynamic Light Scattering (DLS), scanning electron microscopy (SEM) and transmission electron microscopy (TEM). IR spectroscopy measurements were performed using a Thermo Nicolet iS50 FTIR spectrometer equipped with a diamond crystal-based ATR module capable of recording spectra over the range of 400–4,000 cm^−1^. To obtain a better signal-to-noise ratio, the spectra were obtained by summing 16 scans recorded at a resolution of 8 cm^−1^. The acquisition time was set to 3 scans/s using a DTGS type detector and automatic beam splitter for the ability to record the spectrum, especially in transmission mode, in the range 10–25,000 cm^−1^, i.e., from far IR to near IR. The DLS measurements were done using a DELSA Max Pro, light scattering analyzer reusable PEEK flow cells, BCI-3216-DMP, DLS detector angle (degree) 163.5°. The acquisition parameters for this technique were: 5 s acquisition time, 1 s read interval, with 15 s collection period for 3 acquisitions, at 10 Hz electric field frequency, with auto-attenuation set at 0% level, normal laser mode, with set temp at 20° C. SEM images were recorded using a Quanta Inspect F – FEI microscope equipped with an EDX spectrometer. Morphological and structural characterization of AgNPs was published previously by our team (Corbu et al., 2023).

#### 2.2.4 Evaluation of antimicrobial activity against biodeteriogenic strains

##### 2.2.4.1 Microbial strains

Biodeteriogenic strains were previously isolated and identified using classical methods and MALDI TOF MS from Romanian cultural heritage churches (Corbu et al., 2023). For this study we selected two bacterial (i.e., *Bacillus cereus* and *Bacillus megaterium* encoded C16156 and NS5-R) and two filamentous fungi strains (*Penicillium chrysogenum* encoded NS4-2B and NS11-C) previously isolated from wood cultural heritage objects.

##### 2.2.4.2 Qualitative antimicrobial activity of industrial resins

A volume of 10 μL of the four aqueous solutions prepared from the industrial acrylic styrene resin – ADICRIL ARD-795 (1% D1 3 mL AgNPsol, 1% D1 5 mL AgNPc, 0.1% S3 AgNPc and the base coat acrylic resin) was distributed on Sabouraud Dextrose Agar (SDA) in the case of filamentous fungi strains and, respectively, on Mueller-Hinton agar (MHA) for the bacterial strains. After incubation at 37° C for 24 h in the case of bacterial strains, and at room temperature for 6 days in the case of fungal strains, the growth inhibition zone around the disk was measured (mm) (Gheorghe et al., 2021).

##### 2.2.4.3 Quantitative antimicrobial evaluation of industrial resins

The quantitative antimicrobial evaluation of industrial resins and determination of minimum inhibitory concentration (MIC) values were performed as previously described using twofold microdilutions of the screened resins on RPMI (Roswell Park Memorial Institute) 1,640 medium in the case of filamentous fungi strains and Mueller-Hinton Broth (MHB) for bacterial strains in 96 multi-well plates (Corbu et al., 2023).

##### 2.2.4.4 Microbial viability determination in the presence of support materials treated with solutions containing NPs and resins

For wood materials, quantitative evaluation of the antimicrobial activity was evaluated by determining microbial viability in the presence of wood materials treated with siloxane-based coupling agents 3-MPTMS (L-MPTMS) and, respectively, silanization agent + silver nanoparticles (L-MPTMS-Ag100), adapted from previously described methods (Suflet et al., 2024; Adetunji and Odetokun, 2012). Thus, wood fragments with a total area of 1 cm^2^ treated with siloxane-based coupling agents and, respectively, siloxane-based coupling agents + AgNPs were immersed in culture medium (RPMI 1640 for filamentous fungi and MHB for bacterial strains) in a final volume of 2 mL, over which 1/0.5 McFarland suspension of the filamentous fungi/bacterial strains in saline solution, was added. After incubation (6 days at room temperature in the case of filamentous fungi and 24 h at 37° C in the case of bacterial strains) serial decimal dilutions (10^−10^) were inoculated on SDA culture medium (100 μL/dilution) and, respectively, on MH agar (10 μL), followed by incubation (48 h at room temperature and, respectively, 24 h at 37° C). At the end of the incubation period, cell viability was determined as the percentage ratio between the number of colony-forming units (CFU/mL) in each sample (in the presence of functionalized materials) and the CFU/mL obtained in the control (untreated microbial culture).

##### 2.2.4.5 The influence of resins solutions and treated wooden materials on the microbial adherence capacity to the inert substratum

To evaluate the influence of resins solutions on the microbial adherence capacity, the crystal violet microtiter method was used, followed by determination of microbial adherence capacity (Corbu et al., 2024):


$$\text{PICA}\%=\frac{\mathrm{As}\;}{\mathrm{Ac}}\times 100,$$

where As = the absorbance at 490 nm of the biofilm formed and treated with sub-inhibitory concentration of resins solutions,

and Ac = the absorbance at 490 nm of the formed biofilm untreated.

The same protocol was applied for the colonized wood materials after washing three times with saline solution and fixation with methanol. The percentage of adherence capacity inhibition (PICA%) was calculated according to the previous formula, where:

As = absorbance value of the adherence capacity to the treated wooden material (AgNPs and resins and, respectively, wood materials treated with L-MPTMS and L-MPTMS-Ag100);

Ac = absorbance value of the adherence capacity to the untreated material.

To demonstrate the release of antimicrobial compounds into the environment, the effect on the adherence capacity to the surface of the inert substrate was determined in both cases.

##### 2.2.4.6 The influence of wood materials and coatings on extracellular nitric oxide

The extracellular nitric oxide (NO) was quantified using previously reported methods with certain changes (Quinteros et al., 2016). The NO release was evaluated by making a total nitrite spectrophotometric analysis using the Griess reagent. After 24 h of incubation for bacteria and 8 days for fungi suspension, 100 μL of the supernatant was collected and centrifuged at 10,000 rpm for 10 min to remove cells. Then, 50 μL of 2% sulphanilamide in 5% (v/v) H₃PO₄ and 50 μL of 0.1% N-(1-naphthyl)-ethylenediamine aqueous solution were added to 50 μL of supernatant. The mixture was incubated at room temperature for 30 min in the dark. The azo dye was quantified at λ = 540 nm. All measurements were performed in triplicate and results are expressed as mean ± SD. To measure the amount of nitric oxide, a calibration curve with NaNO_2_ values from 1 to 100 μM was created (R2 = 0.9991).

##### 2.2.4.7 The influence of resins solutions on enzymatic and organic acid production

Specific culture media supplemented with cellulose, Tween 20, Tween 80, starch, milk and organic acids were inoculated with treated and untreated microbial suspension followed by the determination of the ratio of the colony diameter (C) to the diameter of the specific inhibition zone occurring around the colony (D) (Corbu et al., 2021):


$$\text{Inhibition}\;(\%)=\frac{D2-C2}{D1-C1}\times 100,$$

where C1—colony diameter of control strain, D1—inhibitory effect zone diameter of control strain, C2—colony diameter of treated strain, and D2—inhibitory effect zone diameter of treated strain.

Microbial strains cultivated in the presence of wooden materials treated with AgNPs and resins and, respectively, wood materials treated with MPTMS and MPTMS-Ag100 were evaluated also regarding the influence against the production capacity to secrete organic acids and enzymes involved in the biodegradation process (cellulase, phenoloxidase, esterase, caseinase and amylase) by cultivation on specific culture media (Corbu et al., 2023). The microbial culture grown in the presence of treated materials was centrifuged 10 min, 10,000 rpm and the cell pellets were re-suspended in saline water to an optical density equivalent to the 1 McFarland standard. The obtained microbial suspensions were seeded spot (10 μL) on the specific agar media. The seeded plates were then incubated for 5 days/24 h at 22/37° C. The influence on the enzymatic capacity and on the production of organic acids was evaluated according to the same relationship used in the case of silanization solution against the enzymatic or organic acid production.

#### 2.2.5 Ecotoxicity of treated wood materials

To evaluate the ecotoxic effects of the wood material functionalized with AgNPs and resins and, respectively, wood materials treated with MPTMS and MPTMS-Ag100, the Allium assay was employed (Corbu et al., 2023; Datcu et al., 2020). *Allium cepa* var. *rubra* bulbs were carefully chosen based on their external morphology and physical soundness. Prior to treatment, all viable bulbs were kept in tap water at ambient temperature for 6 days to encourage root growth. For the control group, bulbs were placed in distilled water, while the experimental groups were exposed to the wood material functionalized with AgNPs and resins and, respectively, wood materials treated with MPTMS and MPTMS-Ag100. After a 24-h exposure period, the bulbs were assessed measuring the fresh biomass by weighing the bulbs on an analytical balance. The data were subjected to statistical analysis using one-way ANOVA with Tukey’s multiple comparisons test, with a single pooled variance, with significance considered at *p* < 0.05.

#### 2.2.6 Statistical analysis

Data are presented as mean ± standard deviation (SD) and the statistical analyses of antibacterial and anti-adherence activities, nitric oxide (NO) content, enzymatic and organic acid production, and ecotoxicity were performed using GraphPad Prism version 9 (GraphPad Software, San Diego, CA, United States). Dunnett’s and Tukey’s multiple comparisons tests were used to correct for multiple comparisons between control and test groups. The significance level was set at *p* < 0.05.

## 3 Results

### 3.1 Synthesis of AgNPs

**Figure 1:**
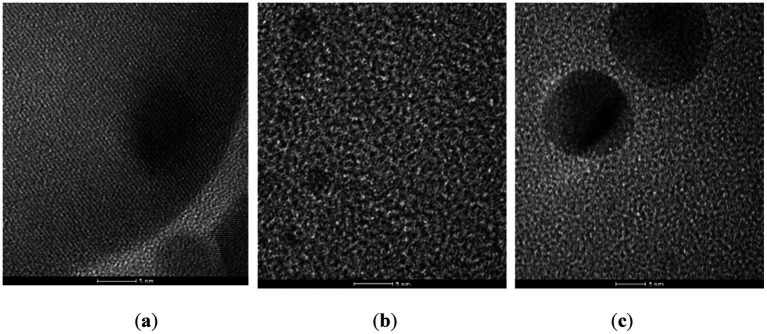
TEM images recorded on AgNPc **(a)**, AgNPt **(b)**, and AgNPsol **(c)**.

The AgNPs were obtained using three different synthesis methods: the classical one, performed at room temperature (AgNPc), Turkevich method, involving reduction in the presence of appropriate agents – AgNPt and the hydrothermal method (AgNPsol); the results of the structural characterization by X-ray diffraction (XRD), FTIR, DLS, SEM and UV–Vis spectroscopy has been published previously (Corbu et al., 2023). AgNPs were in the nanometric range, while they provide the prerequisites and had good stability because the predicted zeta potential is between [−19.60 and −35.45 mV] which leads to a sufficiently high repulsion to keep the suspension stable. In the TEM images (Figure 1) recorded on the three types of AgNPs, it can easily be observed that the size of NPs varies between 10 and 20 nm. By corroborating these data with the DLS, it can be concluded that the particles remain independent, without concerning coalescence.

### 3.2 Characterization of wooden surfaces/materials treated

**Figure 2:**
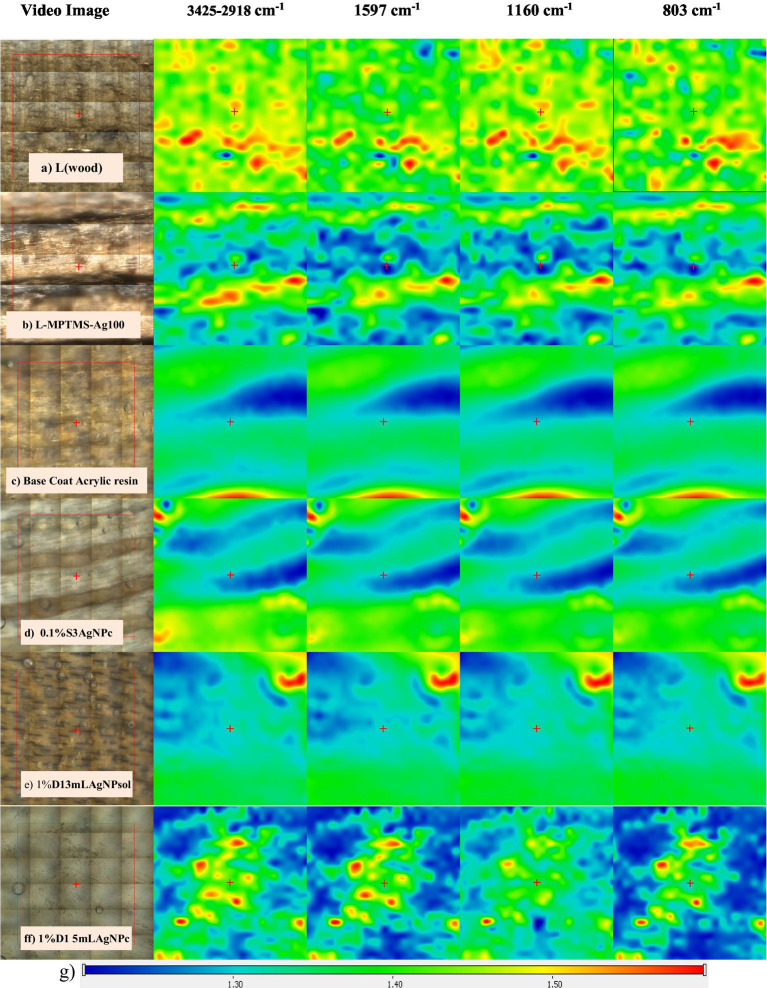
**(a)** FTIR microscopy recorded on (a) wooden model material (L), **(b)** L-MPTMS-Ag100 solutions, **(c)** L-Base coat acrylic resin, **(d)** L-0.1%S3 AgNPc, **(e)** L-1%D1 3mL AgNPsol, **(f)** L-0.1%S3 AgNPc; **(g)** color scale bar to highlight the red to blue differences of the absorbances; for all the reference wood surface and treated surfaces the video image as well as the four FTIR maps are indicated (3425–2,918; 1,597; 1,160 and 803 cm^−1^).

The morphological and structural characterization of the treated surfaces was performed using appropriate physicochemical techniques, specifically FTIR spectroscopy and SEM microscopy. For the wood model materials (L), the surface exhibited a high degree of homogeneity, with identical spectral maps observed across all four monitored wavenumbers. Comparable results were obtained for the samples treated with MPTMS and AgNPs, further confirming that the wood surface was uniformly coated (Figure 2).

**Figure 3:**
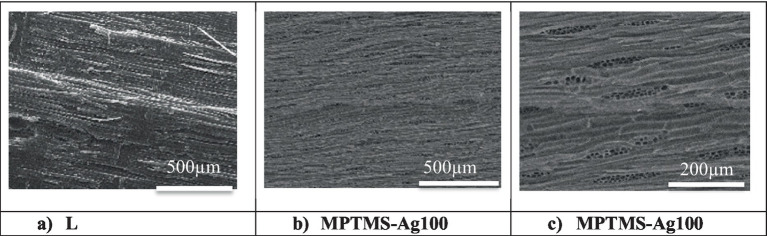
SEM images recorded on wooden model material treated with MPTMS solution containing AgNPs, at different magnifications. **(a)** L. **(b)** MPTMS-Ag100. **(c)** MPTMS-Ag100.

The film morphology and depth penetration were investigated by **SEM microscopy**, at higher magnification. SEM images of untreated and treated samples provided information on the surface appearance. In the case of wood surfaces, SEM images show uniform surface coverage but also an impregnation in the material structure. This coating effect is barely visible in cross-section, but the tangential longitudinal (TL) plane in Figures 3a–c clearly shows transverse pits under the siloxane layer.

**Figure 4:**
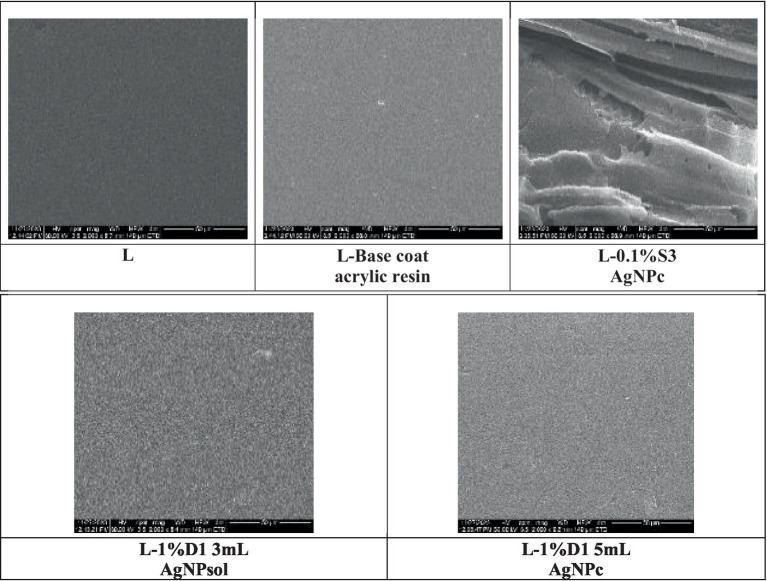
SEM images recorded on wooden model material treated with commercial water-based acrylic resin, Adicril ARD-795, with and without AgNPs.

SEM images of wood surfaces treated with commercial water-based epoxy resin show smooth resin deposits (Figure 4), indicating that the cured resin forms a uniform coating. Both the surface deposits and the coating on the cell walls act as physical barriers against moisture and wood-degrading organisms. While these barriers may initially inhibit the escape of moisture and the intrusion of fungi, they are not entirely impermeable; over time, both moisture and fungal agents may eventually penetrate the protective layers.

**Figure 5:**
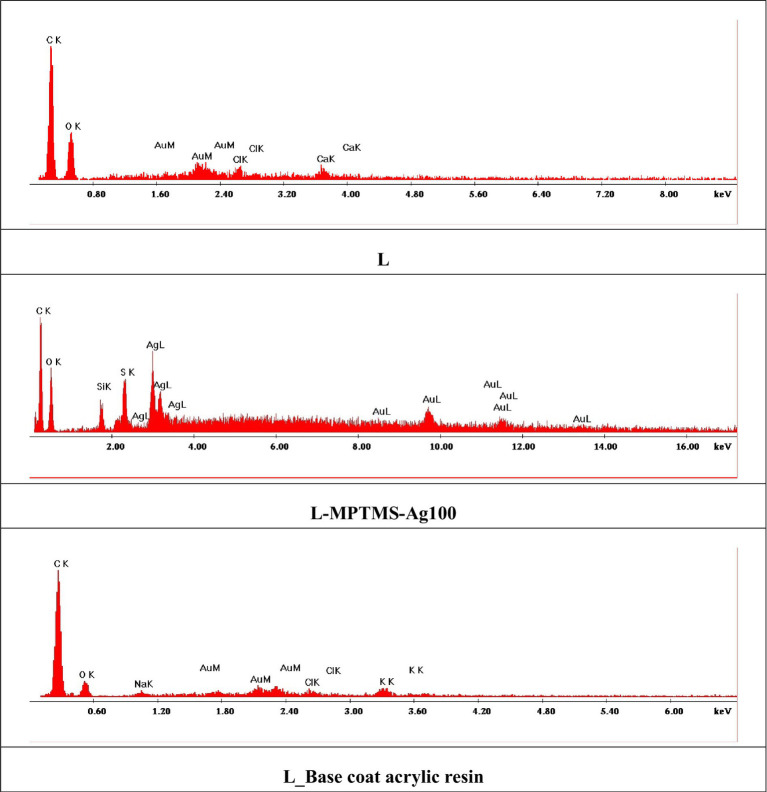
EDX spectra recorded on wooden model material treated with MPTMS or epoxy resin solutions with and without AgNPs solutions.

EDX spectra (Figure 5) confirm the silanization of the surfaces by the presence of the Si, but also that of AgNP. In both cases, these peaks are clearly identifiable, and these elements are not present in the wood reference materials neither in the base coat acrylic resin which means that first, the silanization process was efficient and second, the AgNPs retained well. Considering that MPTMS treatment means a simultaneous surface modification with siloxane and thiol moieties, certainly sulfur is also present, in a relative high amount, the ratio between Si and S being proportional with their atomic weight. These results can be explained considering that MPTMS possess a sensitive hydrolytic center that reacts with organic and inorganic substrates to form stable siloxanic covalent bonds and possess an additional thiolic group with high affinity for silver. In the case of the EDX spectrum recorded on the material treated only with the base coat acrylic resin, a decrease in the intensity of the specific oxygen peak is observed as a result of the coating with acrylic resin while C and O are the major elements and Na as minor element. Au should be ignored being used to metalize the surface for getting better resolution during SEM analyses.

The liquid resin uptake (LU) showed a strong dependency on the type of modification applied. In the case of beech wood, water absorption remained significantly high, with the final mass after immersion increasing by 100–160%. This indicates that treatments with AgNPs and silanization using MPTMS do not effectively reduce water uptake. Nevertheless, these treatments retain important benefits, such as imparting antimicrobial properties, which may contribute to the long-term resistance against microbial attack.

As shown in Supplementary Table S2, a noticeable reduction in water absorption can be achieved through resin treatments. However, such measures are primarily necessary under conditions of prolonged exposure to high humidity or direct immersion in water.

Water absorption after 4 days of immersion did not result in any visible degradation of the experimental wood models. In all cases, the solutions obtained after 96 h showed no detectable reaction to chloride testing for Ag^+^ ions, indicating that ion release was minimal and remained below the technical detection limit.

### 3.3 Antimicrobial activity of the resin coatings and treated wood materials against biodeteriogenic microorganisms

#### 3.3.1 The effect of synthetic resin solutions on the biodeteriogenic microorganism’s growth and metabolic activity

Representative strains, previously characterized as highly relevant as biodeteriogenic microbial models, were selected for determining the impact of synthetic resin solutions on microbial growth and metabolism.

##### 3.3.1.1 Growth inhibition assessment

The qualitative evaluation of the antimicrobial activity of polyacrylic resin solutions revealed that all three tested solutions exhibit an inhibitory effect against the tested bacterial and fungal biodeteriogenic strains after incubation. The antibacterial activity against *B. megaterium* C16156 exhibited a decreasing trend in the following order: 1% D1 3 mL AgNPsol > 1% D1 5 mL AgNPc > 0.1% S3 AgNPc > base coat acrylic resin, indicating the superior efficacy of AgNPs-based formulations, particularly those incorporating AgNPsol.

In contrast, for *B. cereus* C16156, the most pronounced antibacterial effect was observed with the 1% D1 5 mL AgNPc, followed by 1% D1 3 mL AgNPsol, and the base coat acrylic resin, with an arbitrary inhibition unit of 2 (see Supplementary Table S3; Supplementary Figure S1). In the case of fungal strains, the inhibitory areas were no longer detectable after 5 days of incubation.

Following the quantitative assessment of antimicrobial activity, the MIC values against the bacterial strains ranged from 1.56 to 4.08%, indicating moderate to strong antimicrobial effects depending on the formulation. The highest antibacterial efficiency was observed in the case of 1% D1 3 mL AgNPsol (MIC = 1.56%) against *B. megaterium* NS5-R strain, and 1% D1 5 mL AgNPc (MIC = 1.56%) against *B. cereus* C16156 strain. These findings underscore the superior efficiency of AgNP-enhanced coatings compared to both the base acrylic resin and lower-concentration AgNP formulations.

In the case of the fungal strains, the MIC values exhibited a notable variation between *P. chrysogenum* NS4-2B and NS11C strains. For *P. chrysogenum* NS4-2B strain, all formulations including the base coat and AgNP-containing variants demonstrate high MIC values ranging from 20.83 to 25%, suggesting lower susceptibility of this strain. Interestingly, 1% D1 5 mL AgNPc, although the most concentrated, did not enhance antifungal activity and showed the highest MIC (25%), indicating potential strain tolerance. Conversely, *P. chrysogenum* NS11C strain was more sensitive to the AgNP-based formulations, with MIC values ranging from 2.34 to 12.5%. The lowest MIC (2.34%) was recorded for the 1% D1 5 mL AgNPc formulation, followed by 0.1% S3 AgNPc (6.25%) and 1% D1 3 mL AgNPsol (8.33%). The base coat alone exhibited an MIC of 12.5%, further highlighting the enhanced antifungal efficacy of biogenic AgNPs against this fungal isolate. These findings confirm that the antimicrobial efficacy of AgNPs is both formulation and strain-dependent, with higher NP concentrations generally providing improved inhibitory effects, especially against susceptible bacterial and fungal strains (see Supplementary Table S4).

##### 3.3.1.2 Influence on microbial adherence to the inert surface

The anti-adherence potential (PICA%) of the tested resin solutions was evaluated at sub-inhibitory concentrations (MIC/2 and MIC/4) against selected bacterial and fungal strains. Among the bacterial isolates, a notable reduction of the adherence capacity to the inert substratum was observed. For *B. cereus* C16156, the 1% D1 5 mL AgNPc demonstrated the highest anti-adherence effect, with PICA values of 11.92% at MIC/2 and 13.92% at MIC/4 (*p* < 0.0001), indicating a strong inhibitory response. In contrast, the base coat resin showed a moderate effect (58.29% at MIC/2), while the 0.1% S3 AgNPc formulation exhibited no inhibitory activity and even exceeded the control (PICA > 111%), suggesting a potential strain-specific stimulatory response. For *B. megaterium* NS5-R, similar inhibitory trends were obtained. The 1% D1 5 mL AgNPc again achieved the most significant reduction in adherence, with PICA values of 11.96% (MIC/2) and 20.79% (MIC/4) (*p* < 0.0001), while the base resin and 0.1% S3 AgNPc displayed variable and less effective results.

**Figure 6:**
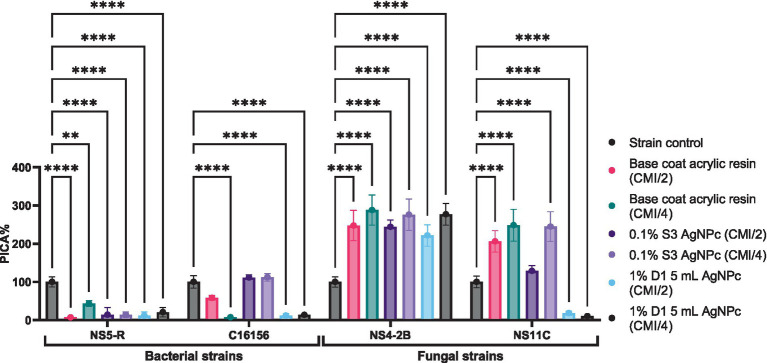
The anti-adherence activity of resin solutions tested against bacterial and microfungal strains (Dunnett’s multiple comparisons test, **p < 0.01; ****p < 0.0001).

In contrast, the fungal strains exhibited divergent responses. For *P. chrysogenum* NS4-2B, all tested formulations including those with AgNPs enhanced adherence capacity relative to the untreated control. Notably, 1% D1 5 mL AgNPc resulted in PICA values of 221.72% (MIC/2) and 277.15% (MIC/4), indicating an undesirable stimulatory effect on adherence and biofilm formation. Conversely, *P. chrysogenum* NS11C responded favorably to the same formulation, with marked inhibition of adherence capacity. The 1% D1 5 mL AgNPc reduced PICA to 18.36% (MIC/2) and 10.65% (MIC/4) (*p* < 0.0001), significantly outperforming both the base resin and the 0.1% S3 AgNPc treatments. In this case, the resin’s anti-adherence efficiency appears to be strain-dependent, highlighting the variable biofilm response mechanisms between fungal isolates (Figure 6) (see Supplementary Table S5).

##### 3.3.1.3 Influence on biodeteriogenic metabolic activity

To evaluate the potential protective role of the tested resin solutions against microbial biodeterioration of cultural heritage objects, was assessed their influence on the production of key extracellular enzymes (esterases, proteases, amylases) and organic acids. These biochemical markers are known contributors to the degradation of organic and inorganic substrates, including historical surfaces. The analysis was conducted at sub-inhibitory concentrations (MIC/2 and MIC/4), and metabolic activity was quantified relative to untreated controls.

**Figure 7:**
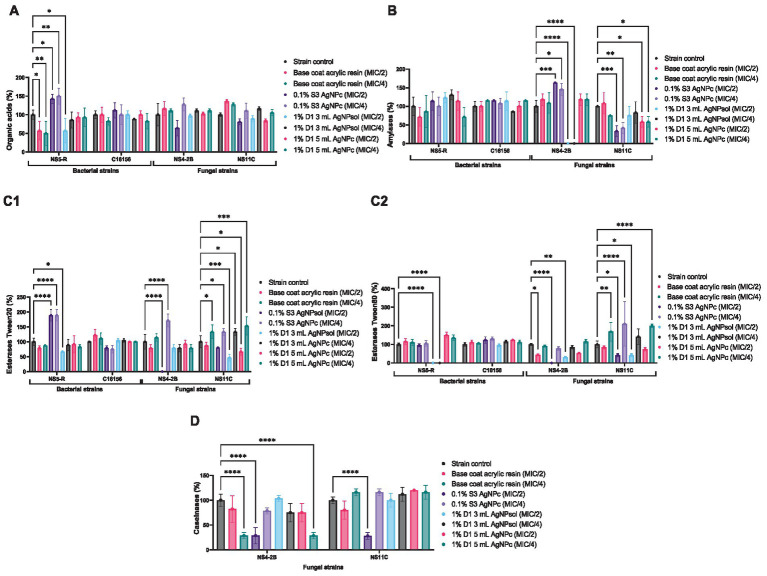
The effect of resin solutions at subinhibitory concentrations on the ability of microbial strains to secrete **(A)** organic acids; **(B)** amylases; **(C)** esterases (1-enzyme substrate Tween 20; 2-enzyme substrate Tween 80), **(D)** caseinases (Dunnett’s multiple comparisons test, *p < 0.05, **p < 0.01; ***p < 0.001, ****p < 0.0001).

In *B. megaterium* NS5-R, exposure to 0.1% S3 AgNPc significantly stimulated organic acid production, reaching 142.86% at MIC/2 and 150% at MIC/4. In contrast, treatment with 1% D1 3 mL AgNPsol and 1% D1 5 mL AgNPc resulted in notable inhibition of acid production, with MIC/2 values of 57.14 and 92.86%, respectively. Esterase activity (targeting ester bonds in long-chain fatty acids) was most effectively suppressed by the 1% D1 3 mL AgNPsol solution, which reduced esterase TW20 production to 66.67% (MIC/2) and 88.89% (MIC/4), and TW80 to 95% (MIC/2) and 115% (MIC/4). The base coat resin showed modest modulation of enzyme levels, while 0.1% S3 AgNPc moderately increased esterase TW20 production to 188.89% (MIC/2), suggesting a possible stress-induced response. Amylase activity, a critical enzyme in polysaccharide degradation, was variably affected, with the 1% D1 3 mL AgNPsol increasing secretion to 123.08% at MIC/2 and 130.77% at MIC/4. In *B. cereus* C16156, the production of all evaluated enzymes and acids demonstrated limited response, with most values approximating 100%, regardless of formulation or concentration (Figure 7) (see Supplementary Table 6). These findings suggest an increased tolerance of this strain to the metabolic modulation effects of the tested coatings.

In *P. chrysogenum* NS4-2B, a clear inhibitory effect was observed in caseinase activity, with 0.1% S3 AgNPc reducing activity to 28.57% at MIC/2, and the base coat acrylic resin and 1% D1 5 mL AgNPc, both exhibiting comparable inhibitory effects at MIC/4. Notably, esterase TW20 and TW80 production was completely suppressed at MIC/2 by the 0.1% S3 AgNPc treatment. Additionally, organic acid production was moderately reduced (to ~63–97%), while amylase activity was strongly inhibited (0% at MIC/2 and MIC/4) by 1% D1 3 mL AgNPsol. In *P. chrysogenum* NS11C, the enzyme production profile differed significantly. The most pronounced reduction in amylase activity was again observed in the case of 0.1% S3 AgNPc (MIC/2–33.33%; MIC/4–41.67%) and 1% D1 5 mL AgNPc solutions (MIC/4–58.33%). Caseinase inhibition was also evident, particularly at MIC/2 in the case of 0.1% S3 AgNPc (28%) and 1% D1 3 mL AgNPsol solutions (75%). Interestingly, this strain demonstrated higher tolerance to esterase inhibition, with values remaining above 40% for all formulations (Figure 7) (see Supplementary Table S7).

These findings indicate that the tested resin solutions, especially 0.1% S3 AgNPc and 1% D1 3 mL AgNPsol, can significantly inhibit the secretion of enzymes and acids involved in microbial biodeterioration, though the magnitude of inhibition is strain- and enzyme-specific. This suggests a promising application of such coatings in the preventive conservation of heritage objects, particularly when tailored to the microbial profile of the treated environment.

##### 3.3.1.4 Extracellular nitric oxide release and correlation with biodeteriogenic features

**Figure 8:**
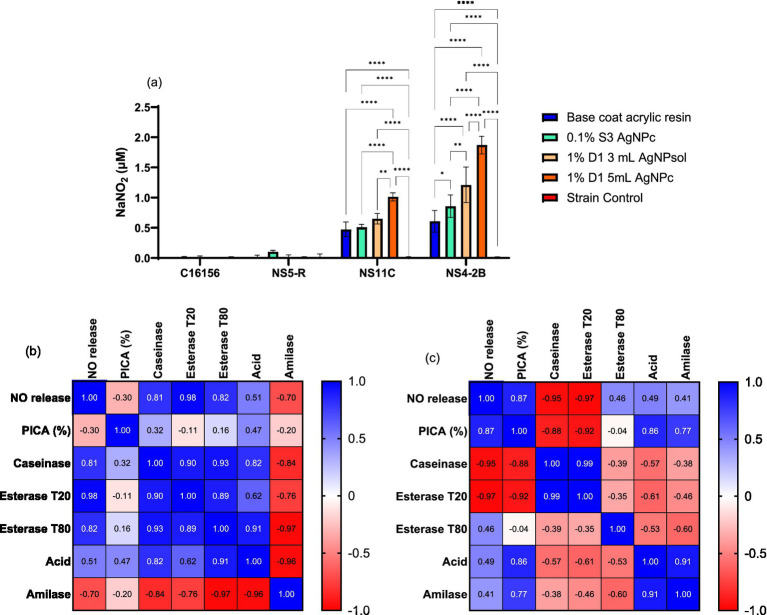
Extracellular NO content determined by Griess’s reaction for AgNPs in presence of biodeteriogenic strains **(a)** (Tukey’s method, *p < 0.05, **p < 0.01, ***p < 0.001, ****p < 0.0001); and Pearson correlation between extracellular NO vs. anti-adherence and enzymatic activities for NS11C strain **(b)** and NS4-2B strain **(c)**.

Considering that one of the ways by which AgNPs might inhibit microbial adherence is the increased NO concentration in the extracellular environment, was studied the effect of a sub-inhibitory AgNPs concentration (MIC/4) on the extracellular release of NO for all tested strains (Figure 8). In our study, it can be observed that extracellular NO for fungal strains was significantly higher than for bacteria, where concentrations of NO were below the limit of detection. The treatment of *P. chrysogenum* NS4-2B strain with MIC/4 of 1% D1 5mL AgNPc exhibited a significantly higher concentration of extracellular NO compared to untreated strain (*p* < 0.0001) as well as to the resin base (*p* < 0.0001) and 0.1% S3 AgNPc (*p* < 0.0001) treatments. The same trend was observed for the *P. chrysogenum* NS11C fungal strain (Figure 8), but with lower values compared to *P. chrysogenum* NS4-2B strain.

In the case of the *P. chrysogenum* NS11C strain, treatment with resin base or AgNPs 0.1% S3 produces moderate growth. The highest values were for the samples 1% D1 3 mL AgNPsol, which showed a significant increase compared to the control and the other treatments (*p* < 0.001), and AgNPs 1% D1 5 mL, which had the highest level of NO for this strain (*p* < 0.0001 compared to control). AgNPs induce the release of NO depending on the type of AgNPs, suggesting an effect dependent on the method of nanoparticle synthesis and dosage.

In the case of *P. chrysogenum* NS4-2B fungal strain, the results were similar to those of the strain NS11C, but the released NO values were higher for all treatments. The treatment with 1% D1 5 mL AgNPc leads to the production of the highest concentration of NO (1.87 ± 0.15 μM NaNO_2_), significantly higher than all other conditions (*p* < 0.0001). All treatments with 0.1% S3 and resin lead to increased levels compared to the untreated control (*p* < 0.0001). Therefore, *P. chrysogenum* NS4-2B strain exhibits the highest capacity for NO release in the presence of AgNPs.

The Pearson correlation was applied for each fungal strain with the following parameters: PICA (%), caseinase, esterase T80, esterase T20, organic acid and amylase. The relationship between extracellular concentration of NO produced at levels of MIC/4 and microbial adherence showed that the higher the NO content, the stronger the adherence inhibition of *P. chrysogenum* NS11C strain (*r* = −0.30) (Figure 8b). In the case of *P. chrysogenum* NS4-2B strain, the correlation was better but with the reverse trend, i.e., the higher the concentration of NO, the better the adhesion (*r* = 0.87) (Figure 8c). In the case of *P. chrysogenum* NS11C strain, direct correlations between NO vs. caseinase activity (*r* = 0.81) and NO vs. esterase’s activity (T20, *r* = 0.98 and T80, *r* = 0.82) have been obtained, i.e., the higher the extracellular content of NO, the better the enzymatic activity. Esterase and protease activity stimulation may be an adaptive mechanism for substrate degradation and the release of nutrients needed for survival in stressful environments. In response to NO, the inhibition of amylase activity could indicate a selective control of enzymatic pathways or a transfer of metabolic resources.

In the case of *P. chrysogenum* NS4-2B strain (Figure 8c), the following correlations can be highlighted: NO content vs. caseinase activity (*r* = −0.95), NO content vs. esterase activity (T20, *r* = 0.97; T80, *r* = 0.46), and NO content vs. PICA (*r* = 0.87). Nitric oxide is a crucial signaling molecule in eukaryotic organisms that controls metabolism and cellular behavior in addition to mediating the stress response. Compared to other fungi, *P. chrysogenum* NS4-2B strain exhibits a different impact on biological processes due to increase NO release, which is most likely brought on by AgNPs. The study found that NO and adhesion capability had a strong positive correlation, suggesting that NO may promote the formation of biofilms or surface contact. NO may encourage ester breakdown, which is essential for fungal metabolism and biodeterioration processes, as it is also strongly linked to T20 esterase activity. Nonetheless, there was a significant inverse relationship between NO and caseinase activity, suggesting a metabolic regulatory mechanism whereby the strain reduces its proteolytic activity in reaction to AgNPs.

#### 3.3.2 Microbial viability and biodeteriogenic activity on treated wood samples

##### 3.3.2.1 Microbial growth

**Figure 9:**
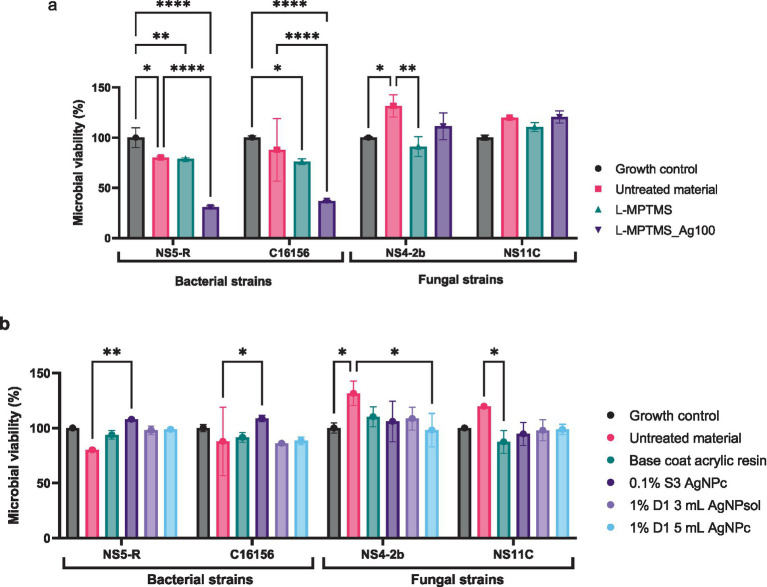
Microbial viability in the presence of wooden material treated with L-MPTMS and L-MPTMS-Ag100 **(a)** and of wooden material treated with synthetic resins **(b)** (Tukey’s multiple comparisons test, *p < 0.05, **p < 0.01, ****p < 0.0001).

The first step of the evaluation involved assessing the viability of microbial strains in the presence of treated materials by quantifying colony-forming units per milliliter (CFU/mL) after 24 h of contact for bacterial strains, and after 6 days for microfungi. As illustrated in Figures 9a,b, the percentage of microbial viability varied depending on both the type of antimicrobial agent applied and the microbial strain tested.

As shown in Figure 9a, treatment of wooden substrate with the silane-functionalized formulation containing AgNPs (L-MPTMS-Ag100), led to a marked and statistically significant reduction in bacterial viability compared to both the untreated control and the growth control. Specifically, the viability percentage of *B. megaterium* NS-5R decreased to 30.66% while for *B. cereus* C16156 the viability was reduced to 36.93% reflecting a robust antimicrobial effect (*p* < 0.0001). This contrasts with the L-MPTMS without AgNPs treatment which exhibited only partial inhibition reducing bacterial viability to 78.78% for *B. megaterium* NS-5R respectively, 76.06% for *B. cereus* C16156 (*p* < 0.05–0.01 depending on the strain). However, the overall antibacterial effect of the treatment is consistent, with differences recorded for the strains analyzed possibly reflecting strain-specific differences in cell wall composition or subtle variations in membrane lipid content, etc.

The fungal strains exhibited a more complex response. For the fungal strain *P. chrysogenum* NS4-2B, exposure to untreated wood led to an increase in viability up to 131.60% likely due to its ability to metabolize lignocellulosic compounds present in wood as carbon source.

Yet, treatment with L-MPTMS alone showed moderate inhibitory effect by reducing with the fungal viability to 91.08% compared to the untreated material (Figure 9a; see Supplementary Tables S8, S9) (*p* < 0.01). The inhibitory effect is likely not due to direct antifungal activity but rather to reduced accessibility of lignocellulosic substrate resulting from increased surface hydrophobicity and chemical modification of the wood surface following L-MPTMS application. Such surface alteration may hinder fungal adhesion and enzymatic degradation processes, thereby limiting fungal proliferation. In case of *P. chrysogenum* NS11C strain no significant alteration of fungal viability were recorded proving once again that the impact of L-MPTMS alone or supplemented with AgNPs is strain dependent.

As shown in Figure 9b the base coat acrylic resin failed to reduce microbial viability appreciably across the strains tested. However, adding AgNPs improved the outcomes. For *B. cereus* C16156 both 1% D1 AgNP solutions formulated with 3 or 5 mL of AgNPs led to significant reductions in viability to 86.19% and 88. 43% (*p* < 0.01). Fungal strains demonstrated greater resistance to the applied treatments. For the fungal strain, all tested acrylic resin formulations led to a decrease in microbial viability relative to the untreated wood (131.60 and 119.78%), though the reductions were not statistically significant when compared with the growth control (see Supplementary Tables S8, S9). Notably, the 1% D1 5mL AgNPc formulation showed the greatest antifungal effect among resin treatments, though the overall viability remained above 80% for both fungal strains. Interestingly, a reduction of NS11C viability compared to the untreated substrate was observed when base coat acrylic resin treatment was applied probably due to the inaccessibility of nutrients from the wooden material. Overall, the results indicate that AgNPs enhance the antimicrobial properties of both silane- and resin- based wood treatments, with bacterial strains being more susceptible than fungal strains.

##### 3.3.2.2 Adherence capacity of the biodeteriogenic strains

**Figure 10:**
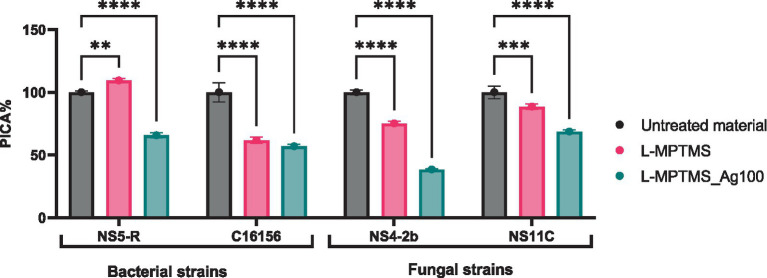
The inhibitory effect of treated wood material on the adhesion capacity of biodeteriogenic bacterial and fungal strains (Dunnett’s multiple comparisons test, **p < 0.01, ***p < 0.001, ****p < 0.0001).

Based on the results obtained for microbial viability, the study continued with determination of the impact of the treatments on the microbial adherence capacity. As shown in Figure 10, treatment of wood with L-MPTMS and the AgNPs enhanced L-MPTMS significantly reduced microbial adhesion of both bacterial and fungal strains (compared to the untreated material (*p* < 0.0001 for most comparisons). The most substantial reductions were recorded in case of *B. cereus* C16156 and *P. chrysogenum* NS4-2B with PICA% values decreasing to 61.78 and 56.93%, respectively, following L-MPTMS treatment, and further down to 75.24 and 38.43% when treated with L-MPTMS-Ag100 (see Supplementary Table S8). These results confirmed the above-mentioned statement regarding the fact that the treatment with L-MPTMS alters the physiochemical properties of wood surfaces in terms of hydrophobicity or surface roughness thereby impairing microbial attachment. Adding AgNPs in the L-MPTMS-Ag100 formulation appears to amplify this anti-adhesive effect, possibly through a combination of surface modification and the well-documented antimicrobial activity of AgNPs which can inhibit microbial surface colonization at early stages. Notably, the fungal strain *P. chrysogenum* NS4-2B exhibited a strong response to the AgNPs enhanced treatment, with PICA% reduced nearly by half.

**Figure 11:**
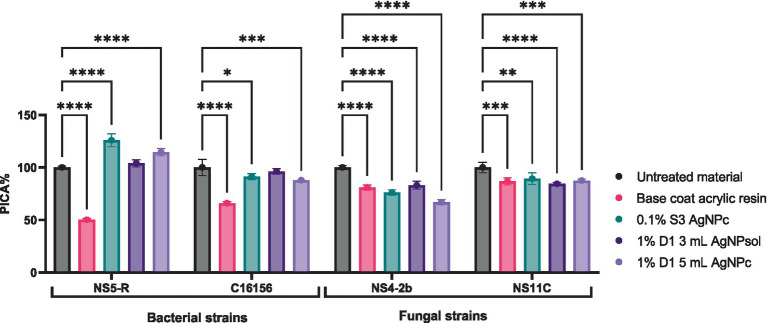
The inhibitory effect of wood material treated with industrial resins on the adhesion capacity of bacterial strains and filamentous fungal strains (Dunnett’s multiple comparisons test, *p < 0.05, **p < 0.01, ***p < 0.001, ****p < 0.0001).

The evaluation of microbial adherence capacity to resin-treated wooden materials revealed that the base coat acrylic resin treatment exhibited the most pronounced anti-adhesive effect among the tested industrial resins. Specifically, a significant reduction in surface colonization was observed for *B. megaterium* NS5-R, with PICA% decreasing up to 50.13%. However, adding AgNPs significantly reduced the adhesive capacity for 3 out of 4 strains, while for the fourth one, *B. megaterium* NS5-R, an increase of the PICA% value was observed (114.58 in case of 1% D1 5mL AgNPc respectively, 125.97% in case of 0.1% S3 AgNPc) (Figure 11, see Supplementary Table S9).

**Figure 12:**
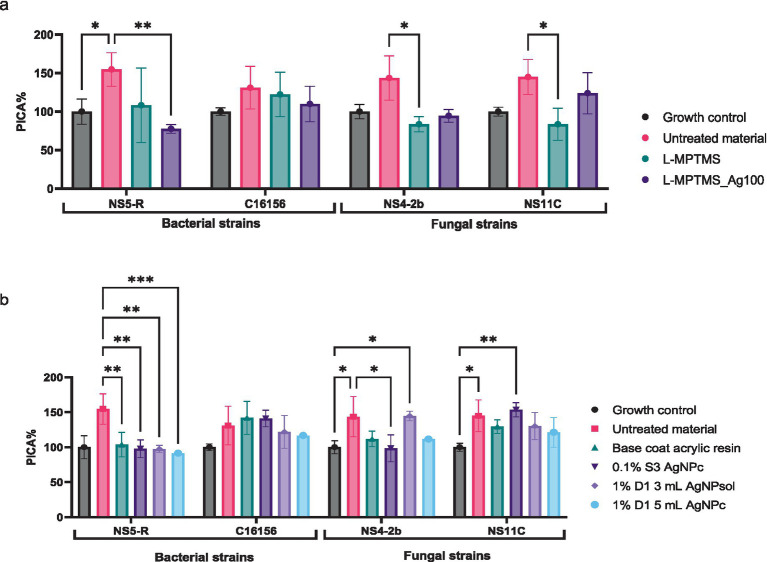
The inhibitory effect of compounds released from wood materials type **(a)** and wood materials type treated with acrylic resins **(b)** on the adhesion capacity of bacterial and filamentous fungal strains to the inert substrate (Tukey’s multiple comparisons test, *p < 0.05, **p < 0.01, ***p < 0.001).

Since large quantities of active antimicrobial compounds can be released from the substrate in the presence of high humidity, the study continued for determining the impact of these released compounds on the adherence capacity to other substrates. According to Figure 12, no significant changes in the adherence capacity of bacterial strains to the inert substratum as a result of exposure to the antimicrobial compounds released have been noticed. In the case of the fungal strains, a decrease in the adherence capacity compared to the untreated material presence has been observed. Thus, it can be concluded that some of the antimicrobial compounds used to treat surfaces reach the culture medium and interfere with the metabolism of non-adherent cells, inhibiting their ability to further colonize the surface. In case of wood material treated with MPTMS the adhesion of *B. megaterium* NS5-R to the inert substratum was moderately inhibited by the L-MPTMS_Ag100 formulation, resulting in a PICA% of 77.62%. Furthermore, *B. cereus* strain C16156B adhesion to the inert substratum was enhanced, with PICA% values ranging from 109.86 to 130.96%. Fungal adhesion capacity was not significantly inhibited, with only a slight reduction observed for the L-MPTMS material, showing PICA% values around 83% (Figure 12a, see Supplementary Table S8).

In contrast, regarding the wood material treated with acrylic resins in the presence of NS5-R *B. megaterium*, compared to the untreated material, all treatments incorporating 0.1% S3 AgNPc, 1% D1 3 mL AgNPsol, 1% D1 5 mL AgNPc acrylic solutions slightly inhibited the capacity to adhere to the inert substratum with PICA% values around 91%. Meanwhile, for the *B. cereus* C16156 strain, surface adhesion was stimulated in the presence of all the tested materials, with PICA% values surpassing 116%, suggesting limited inhibitory effects. For *P. chrysogenum* NS4-2b and NS11C fungal strains, the adhesion capacity remained generally unaffected by most treatments. However, a modest inhibitory effect was observed for NS4-2b strain, particularly after treatment with 0.1% S3 AgNPc when PICA% values decreasing to approximately 98% (Figure 12b, see Supplementary Table S9).

##### 3.3.2.3 Biodeteriogenic metabolic features

When exposed to the treated materials it was observed that the AgNPs solution retained its functional bioactivity by significantly reducing the secretion of organic acids by *P. chrysogenum* NS4-2B, with up to 30% while for the *P. chrysogenum* NS11C a similar effect was observed in case of esterase activity (up to 60% inhibition). More precisely in the case of bacterial strains exposed to the AgNPs coating solution completely inhibited the esterase activity of *B. megaterium* NS 5-R strain and up to 30% for the *B. cereus* C16156 strain (see Supplementary Table S10). These results suggest that AgNPs maintain their impact on key metabolic pathways even after being applied on the wooden surface as functionalized coatings. Beyond esterase and organic acid production, further enzymatic assays demonstrated that AgNP exposure also impaired the activity of other extracellular enzymes critical for microbial survival and competitiveness. Specifically, cellulase production was completely inhibited in the presence of L-MPTMS in case of *B. megaterium* NS5-R and *B. cereus* C16156 but highly simulated when AgNPs were added (550% respectively, 180%). Overall, the results indicate that proteolytic and cellulolytic functions are influenced by the treatment based on L-MPTMS with or without AgNPs mediated stress. Although amylase activity showed variable susceptibility, certain isolates exhibited significant reductions, suggesting strain-specific sensitivity possibly linked to differences in cell wall structure or enzyme regulation.

Treating the surface of the wood material with the resin solution has a minor impact on the ability of microbial strains to secrete enzymes or organic acids. However, a pronounced inhibitory effect was observed in the case of the strain *P. chrysogenum* NS11C whose ability to secrete caseinase and organic acids, respectively, was inhibited in the presence of material treated with 0.1 S3 2020 solution (71.42 and 70.49%). Similarly, the ability of *B. cereus* strain C16156 to secrete esterase when cultured in the presence of 1%D1 5mL AgNPs treated material was reduced to 54.55% (see Supplementary Table S11). Beyond these prominent examples, other strains exhibited varying degrees of sensitivity across enzymatic functions. Moderate reductions were observed in amylase activity in case of bacterial isolates, highlighting that while the inhibition is not universal, multiple enzymatic pathways can be impacted depending on the treatment composition, concentration and microbial target. The amylase production was severely reduced in presence of 1% D1 5mL AgNPc (to 39.39%) for *B. megaterium* NS5-R strain but no significant impact was recorded for the *B. cereus* C16156 strain.

##### 3.3.2.4 Extracellular NO release and correlation with biodegradative features

**Figure 13:**
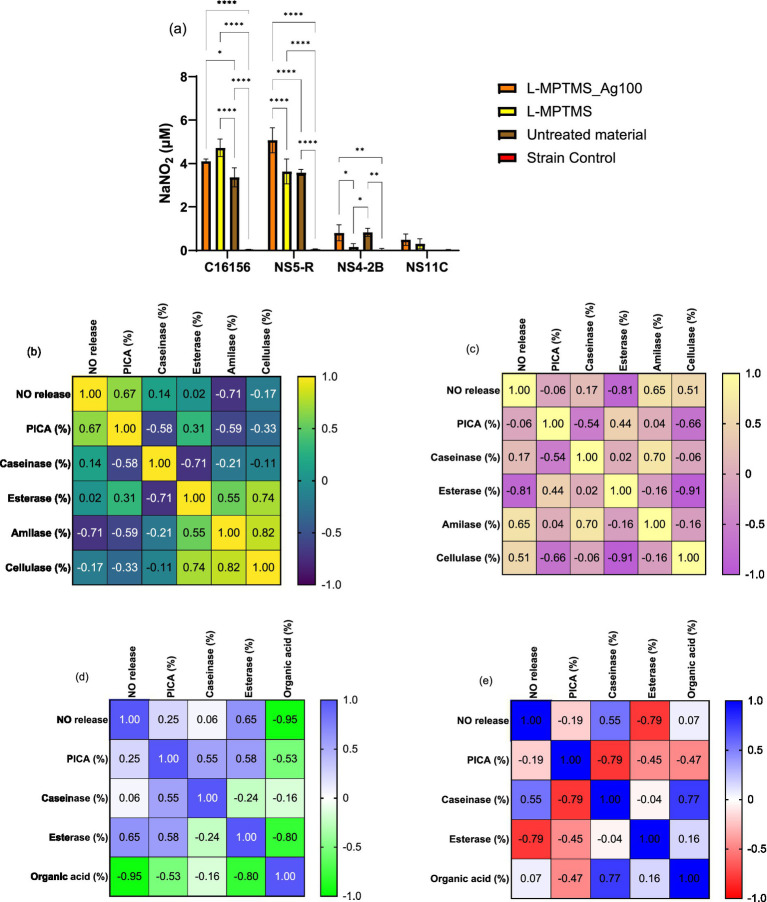
Extracellular NO content determined by Griess’s reaction for the biodeteriogenic strains in presence of silanized wood materials **(a)** (Tukey’s method, *p < 0.05, **p < 0.01, ***p < 0.001, ****p < 0.0001); and Pearson correlation between extracellular NO vs. anti-adherence and enzymatic activities for C16156 **(b)**, NS5-R **(c)**, NS4-2B **(d)** and NS11C **(e)** strains.

According to the data in Figure 13a, the addition of silver ions (L-MPTMS_Ag100) decreases this response (*p* < 0.05), possibly due to an antimicrobial effect; in contrast, *P. chrysogenum* NS4-2B and NS11C fungal strains showed very low levels of NO, with no significant differences between treatments. The wood material, particularly the silane-functionalized one (L-MPTMS), significantly stimulates the extracellular release of NO in the case of bacterial strains (*B. cereus* C16156 and *B. megaterium* NS5-R) when compared to the untreated material (*p* < 0.0001). The NO release demonstrated how treatments applied to wood surfaces impact microbial physiology, and the differences between bacteria and fungi highlight how specific the response is depending on the type of microbe and the properties of the surface. Therefore, keeping an eye on NO can be a helpful way to assess how well antimicrobial treatments work on porous materials like wood.

In the case of *B. cereus* C16156 strain, the correlations with esterase and caseinase activities were not significant (Figure 13b). Instead, the activity of cellulase and amylase decreases, and the inhibition of adhesion increases with NO content increase. It is noticeable that, in the case of *B. megaterium* NS5-R bacterial strain, high concentrations of NO inhibit only esterase activity (Figure 13c). Regarding *P. chrysogenum* NS4-2B strain, the acidity decreased with increasing the NO content, which may indicate a decrease in the degree of wood material degradation caused by acid hydrolysis (Figure 13d). Enzyme inactivation such as esterase activity (*r* = −0.80) and enhanced PICA (*r* = −0.53) might also result from the acid pH. The variations in enzymatic activity and microbial adhesion capacity recorded for *B. megaterium* NS5-R strain were different (Figure 13e) indicating that these correlations are highly dependent on the microbial strains under investigation. NO can serve as either a stimulant or an inhibitor, influencing processes such as substrate adherence, polymer breakdown, and organic acid secretion. Increased NO release in bacterial strains such as *B. cereus* C16156 may limit carbohydrate breakdown and surface adherence, potentially through oxidative stress defense mechanisms. *B. megaterium* NS5-R responds differently, blocking esterases while boosting amylase activity, indicating a distinct metabolic response to NO. A negative association between NO and extracellular acidity in filamentous fungi may imply a barrier in organic acid secretion, which reduces the effectiveness of wood material decomposition. The diversity in *P. chrysogenum* NS11C response indicates its high metabolic complexity and versatility.

**Figure 14:**
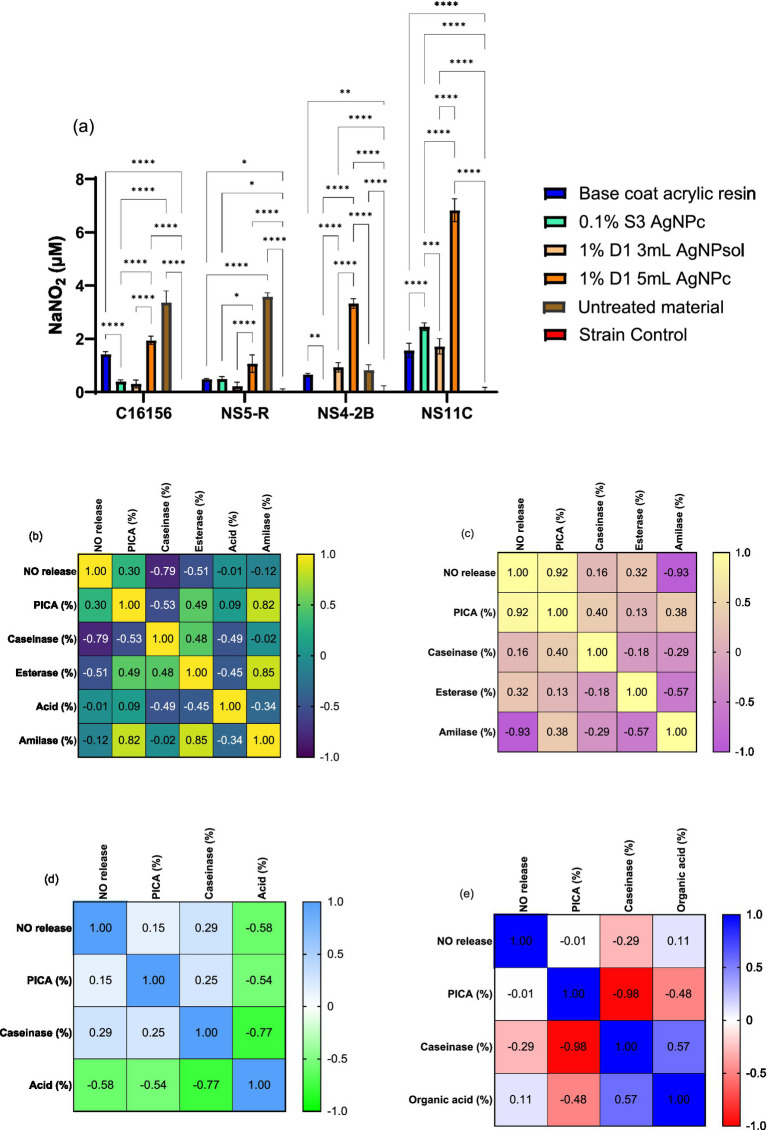
Extracellular NO content determined by Griess’s reaction for the biodeteriogenic strains in presence of wood materials coated with resins **(a)** (Tukey’s method, *p < 0.05, **p < 0.01, ***p < 0.001, ****p < 0.0001); and Pearson correlation between extracellular NO vs. anti-adherence and enzymatic activities for C16156 **(b)**, NS5-R **(c)**, NS4-2B **(d)** and NS11C **(e)** strains.

In the case of resins, it can be observed that the treatment base can significantly change the behavior of biodeteriogenic microorganisms. Thus, in the case of bacterial strains a significant decrease in NO has been observed compared to untreated material, practically the treatment leading to the protection of cells from nitrosative stress. In the case of fungi, it has been observed that the 1%D1 5 mL AgNPc treatment leads to a significant increase in the extracellular NO content (Figure 14a). The Pearson correlation showed that the higher the NO content, the lower the enzymatic activity for amylase, esterase and caseinase. For the bacterial strain *B. cereus* C16156 (Figure 14b), an increase in extracellular NO content was associated with a strong inhibition of caseinase activity (*r* = −0.79), whereas amylase activity correlated positively with microbial adhesion (*r* = 0.82), implying that this enzyme may play a functional role in the colonization process. Furthermore, the acidity significantly influences the activity of these enzymes, even though it is not directly correlated with the NO content. In the case of *B. megaterium* NS5-R strain (Figure 14c), the Pearson correlation showed that the activity of amylase was decreased as the concentration of NO increases. In the case of this strain, a very good correlation with PICA (%) can be observed, in the sense that the higher the NO content, the greater the microbial adhesion. In the instance of *B. megaterium* NS5-R strain (Figure 14c), high doses of NO greatly favored adhesion (PICA, *r* = 0.92), but strongly hindered amylase activity (*r* = −0.93), indicating an adaptive response in which the cell prioritizes attachment over degradation activities.

For *P. chrysogenum* NS4-2B strain, it can be observed that the acidity is inversely proportional to the extracellular content of NO and PICA, having the ability to inactivate caseinase (Figure 14d). A negative correlation between NO and extracellular acidity (*r* = −0.58) indicates that nitric oxide reduces the acidic environment, which favors proteolytic activity (caseinase), given that higher acidity correlates negatively with it (*r* = −0.77). In the case of *P. chrysogenum* NS11C strain, the increase in acidity can lead to a reduction in microbial adhesion (Figure 14e). *P. chrysogenum* NS11C fungal strain had lesser correlations, but there was a very significant negative association between microbial adherence and caseinase activity (*r* = −0.98), indicating a possible metabolic trade-off between colonization and extracellular enzyme synthesis.

Overall, the findings showed that NO release affects microbial responses differently depending on the strain, inhibiting hydrolytic enzymatic activity while also influencing adhesion and extracellular acidity. NO interferes with biofilm development and can disperse established biofilms through cell signaling pathways at low levels (10^−9^–10^−6^ mM). High NO concentrations (0.001–1 mM) remove biofilms by directly killing embedded bacteria (Yang et al., 2018).

### 3.4 Ecotoxicity

**Figure 15:**
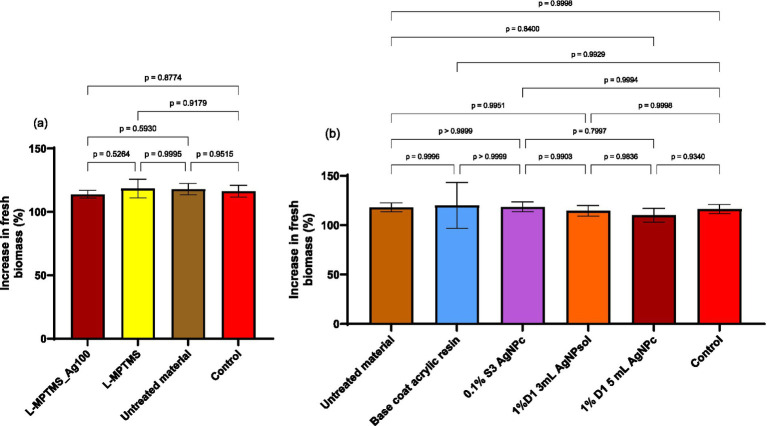
Comparative results for the increase in fresh biomass of *A. cepa* bulbs after 24 h: **(a)** wood-based materials coated with silane-based coatings, and **(b)** wood-based materials coated with acrylic resins. Statistical analysis method used was one-way ANOVA with Tukey’s multiple comparisons test, with a single pooled variance (significant relevance was set *p < 0.05).

The main objective of the ecotoxicity test in this study consists in assessing the short-term impact on the bulbs of *A. cepa*, i.e., for 24 h, after direct contact. In terms of fresh biomass, the weight of *A. cepa* bulbs increased across all variants. Thus, Figures 15a,b reveal that none of the coating solutions were significantly harmful related to the control. Coating with resin 1%D1 5 mL AgNPc resulted in the lowest biomass growth value when compared to the control, although this was not statistically significant (*p* > 0.05) (Figure 15b). In the case of silane coatings, the L-MPTMS-Ag100 variation resulted in a modest decrease in *A. cepa* bulb biomass, although the *p*-value was higher than 0.05 which is beneficial and can be attributed to the high affinity of the thiol groups to the AgNPs surface. It may be stated that the solutions provided do not exhibit phytotoxicity.

The increase in fresh biomass across all tested samples suggests that the applied materials, whether acrylic resins with AgNPs or silane-treated materials, did not cause a significant stress response on plant metabolism throughout the 24 h exposure period. Surface interactions, such as the affinity of thiols in silanes for AgNPs, can influence the degree of Ag^+^ ion release, as seen in L-MPTMS-Ag100 and 1% D1 AgNPc. However, the lack of significant decreases in fresh biomass supports the notion that these materials do not exhibit acute phytotoxicity and are thus compatible with plant biological media under similar testing conditions.

## 4 Discussion

This study explored the antimicrobial and protective efficacy of treatments combining commercial polyacrylic resins, silver nanoparticles (AgNPs), and siloxane-based coupling agents (3-MPTMS) for the conservation of wooden heritage materials. Polyacrylic resins are widely used as surface coatings due to their advantageous properties, including rapid drying time, optical clarity, favorable mechanical strength, strong adhesion, and excellent chemical stability (Cocca et al., 2004). Protective treatments employing commercially available acrylic resins are not only easy to apply and cost-effective, but also non-destructive to the treated surfaces. These resins are capable of penetrating the substrate, providing structural reinforcement and enabling deep impregnation, particularly in microcracks and fissures, rather than forming a superficial coating alone. The consolidation of wooden elements using resins is well documented, for instance in the repair of wooden elements in Nanzen Temple in Shanxi (Wang et al., 2023), as well as in the restoration of ancient architectural heritage in Brazil and Spain (Da Silva Bertolini et al., 2013; Vilches Casals et al., 2013).

Other polymer-based systems such as FEVE resins modified with Ag2O/OH-MWCNTS have also shown enhanced viscosity, thermal stability, and antimicrobial activity against *Trichoderma* spp., *Aspergillus niger*, and white rot fungi (Teri et al., 2024). Similarly, the use of polyacrylic resins enhanced with AgNPs and siloxane-based coupling agent 3-MPTMS represents a potent strategy for combating microbial contamination and preserving materials susceptible to biodeterioration.

The role of the coupling agent is to create a bond between the silicon in the siloxane groups (Si-MetOH) and the -OH hydroxyl groups on the structure of the substrates. The siloxanes used possess a hydrolytic center that reacts with inorganic substrates to form stable covalent bonds and an organic group that modifies the physical interactions of the treated substrates. Through hydrolysis and condensation reactions, alkoxy groups of siloxanes eliminate water or alcohol to form a crosslinked network, partially shrinking the pores of the treated surface through the pore-lining effect (Marinescu et al., 2023). Terminal –SH moieties on the siloxane molecules covalently bind to AgNPs, promoting self-assembly and enhancing penetration into wood structure.

Compared to other coating systems, surface silanization methods are easy to apply, cost-effective, non-destructive, and compatible with solvent systems (especially alcohol-based ones) that facilitate faster evaporation and deeper substrate penetration. Siloxane-based coupling agents are proposed as alternatives to benzotriazole due to their hydrophobicity, transparency, and corrosion-inhibiting properties, making them suitable for cultural heritage conservation (Artesani et al., 2020). Silane coatings interact covalently with metal surfaces when hydrolyzed, though achieving uniform films requires multiple layers depending on substrate properties (Schreiber, 2000).

The combined application of AgNPs with silanization agents enhances antimicrobial efficacy and mechanical reinforcement. Silane-modified epoxy resins, for example, improve tensile strength and reduce water absorption in viscose fabric composites (Rajan et al., 2018). Similarly, advanced mortar coatings treated with silanization agents have demonstrated exceptional durability under UV exposure, offering long-term protection for historical buildings (Jelincic and Glivetić, 2020). Nonetheless, high NP concentrations may alter the visual appearance of treated substrates, requiring optimized formulations (Ntelia and Karapanagiotis, 2020).

In our study, treatments combining AgNPs with MPTMS or acrylic resins significantly reduced microbial viability, particularly for bacterial strains such as *Bacillus cereus* and *Bacillus megaterium*. This finding aligns with prior research showing that AgNPs disrupt microbial membranes, produce reactive oxygen species, and damage DNA (Rai et al., 2009; Seth et al., 2012). Notably, untreated wood enhanced the growth of *P. chrysogenum*, emphasizing the biodeteriogenic potential of fungal colonization.

Sub-inhibitory concentrations of resin solutions inhibited bacterial adherence to inert substrata, with PICA% values significantly lower than controls. This anti-adherence activity was accompanied by increased oxidative stress, as evidenced by elevated extracellular nitric oxide (NO) levels, particularly in fungal strains. These findings support the hypothesis that NO plays a role in microbial stress responses and biofilm inhibition (Bowman et al., 2011; Yang et al., 2018). In contrast, bacterial strains produced lower extracellular NO, and AgNPs decreased NO production while MPTMS increased it, suggesting distinct nitrosative stress responses (Stern and Zhu, 2014).

Enzymatic activity was also influenced by treatment type and microbial strain. The strongest inhibition of esterase and amylase secretion was observed for fungal strains treated with 0.1% S3 and 1% D1 AgNPs. Bacterial esterase activity was completely inhibited in *B. megaterium* and significantly reduced in *B. cereus*. These data support the dose-dependent, species-specific effects of AgNPs on microbial enzymatic systems (Quinteros et al., 2016).

The potential ecological impact of these treatments was assessed using the *Allium cepa* test system. No significant phytotoxic effects were observed, as all treated samples supported normal biomass growth. This confirms the environmental safety of these formulations, addressing concerns about the release of AgNPs from treated surfaces (Ameen et al., 2021; Souza et al., 2020).

Regarding NO, its multifunctional role in microbial physiology was confirmed. While some microbial strains increase NO production as a defensive response, others reduce it under AgNP exposure. This differential response influences not only microbial adhesion but also enzymatic activity and degradation potential. Studies have shown that NO interacts with microbial enzymes, potentially inactivating them via S-nitrosylation and impairing pathogenicity (Brandes et al., 2007; Stern and Zhu, 2014).

The study was further extended to evaluate the inhibitory efficacy of resin solutions applied to model materials, specifically 1 cm^2^ wood fragments. Additionally, the analysis included a wood model pre-treated with MPTMS and subsequently functionalized with AgNPs synthesized via the Turkevich method (Ag100), which demonstrated high levels of antimicrobial activity in a previous study (Marinescu et al., 2022). The wood model materials treated with L-MPTMS and L-MPTMS-Ag100 decreased the microbial adhesion and enzymatic degradation. Our results are in agreement with a previous study showing that organosilicon compounds can preserve degraded wood by reinforcing structure and protecting against further biodeterioration (Broda et al., 2023; Broda and Plaza, 2023). The same effect was reported for 3-MPTMS and AgNPs deposited on other substrates like cotton fabric, enhancing its antimicrobial activity (Kim et al., 2011).

Similarly, the most effective resin formulation, 1% D1 5 mL AgNPc, strongly inhibited microbial adherence and enzymatic activity, supporting its suitability for conservation use. This aligns with findings on AgNP-embedded catechol-formaldehyde resin microspheres and other antimicrobial coatings that inhibit bacterial and fungal growth with low toxicity (Liu et al., 2024).

Finally, it is essential to consider the long-term fate of nanoparticles. While AgNPs show great promise in conservation, their ecological and toxicological profiles must be closely monitored. Existing literature highlights the potential for AgNPs to affect soil and plant microbiomes, but our findings show minimal short-term phytotoxicity and support their use in responsible, controlled conservation applications (Cvjetko et al., 2017; Sajid et al., 2015).

## 5 Conclusion

This study demonstrated that polyacrylic resins incorporating AgNPs possess substantial antimicrobial properties, with notably stronger effects against bacterial strains. The incorporation of AgNPs, particularly in combination with MPTMS or polyacrylic matrices, significantly enhanced the inhibition of microbial viability, adhesion, and the secretion of degradative enzymes. Additionally, sub-inhibitory concentrations of resin-based treatments modulated extracellular NO levels, indicating activation of microbial stress responses. Importantly, ecotoxicological evaluations confirmed that these functionalized materials exhibit minimal phytotoxicity, supporting their environmental safety and suitability for use in the conservation of cultural heritage. Furthermore, treatments applied to wood model substrates, especially L-MPTMS and L-MPTMS-Ag100, effectively reduced microbial adhesion and enzymatic degradation, reinforcing their protective potential for organic heritage materials. These findings support the application of AgNP-functionalized polyacrylic resins as safe and effective antimicrobial coatings for the preservation of cultural heritage objects, with both immediate and long-term implications for biodeterioration control strategies.

Future studies should focus on assessing the efficacy of these materials against a broader range of biodeteriogenic taxa, including white rot fungi and erosion bacteria, within more complex microbial communities. In addition, omics-based analyses and molecular-level investigations (e.g., transcriptomics, proteomics, and metabolomics) could provide deeper insight into antimicrobial mechanisms and stress pathways. Lastly, long-term performance and environmental stability of these treatments should be evaluated under simulated and real environmental conditions, including fluctuations in temperature, humidity, UV exposure, and microbial succession. Monitoring aging behavior, leaching potential, and re-treatability will be essential for validating the materials’ safety, durability, and compatibility with conservation ethics and standards.

## Data Availability

The original contributions presented in the study are included in the article/Supplementary material, further inquiries can be directed to the corresponding authors.
