# Supplementary material for: Multifunctional protective strategies for wooden cultural heritage: antimicrobial efficacy of polyacrylic resins, siloxane coupling agents, and silver nanoparticles
Source: Front Microbiol. 2025 Aug 22;16:1642335. doi: 10.3389/fmicb.2025.1642335 (PMC12411555; doi:10.3389/fmicb.2025.1642335)
Supplement: Supplementary file 1 [file Supplementary_file_1.docx]

Supplementary Material

**Multifunctional Protective Strategies for Wooden Cultural Heritage: Antimicrobial Efficacy of Polyacrylic Resins, Siloxane Coupling Agents, and Silver Nanoparticles**

Andreea Ștefania Dumbravă^1,2^ ^†^, Viorica Maria Corbu^3,4^ ^†^, Ioana Cristina Marinaș^3^‡, Radu Pericleanu^1^, Liliana Marinescu^5^, Ludmila Motelica^6,7,8^, Doina Roxana Trusca^8^, Nicoleta Ianovici^9^, Tatiana Eugenia Șesan^1,10^, Irina Gheorghe-Barbu^1,3^***‡**, Denisa Ficai^5,7,8§^, Ovidiu Cristian Oprea^5,7,8^, Anton Ficai^5,7,8^, Mariana Carmen Chifiriuc^1,3,7§^

*** Correspondence: irina.gheorghe@bio.unibuc.ro**

^†^ These authors contributed equally to this work and share first authorship

‡ These authors contributed equally to this work and share the corresponding authorship

§ These authors have equal contributions and share the last author position

# Supplementary Figures and Tables

## Supplementary Figures


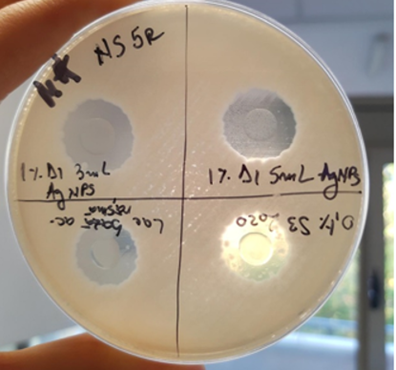

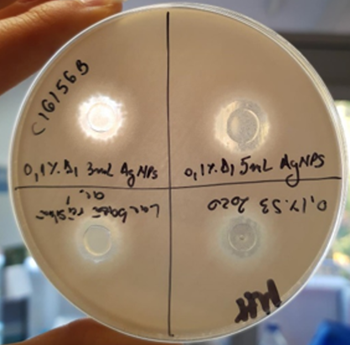


**A**

**B**

**Supplementary Figure 1.** Disk diffusion screening assays of the antifungal activity of the tested polyacrylic resin solutions against bacterial strains (1% D1 3ml AgNPsol, 1% D1 5ml AgNPc, 0.1% S3 AgNPc, Base coat acrylic resin) against A- NS 5R *Bacillus megaterium* and B- C16156B *Bacillus cereus*.

## Supplementary Tables

**Supplementary Table 1.** Composition of the Base coat acrylic resin with AgNP samples.

| Type sample | Base coat  acrylic resin | AgNPc | AgNPsol | Distilled water H_2_O |
| --- | --- | --- | --- | --- |
| Base coat  acrylic resin | 78 | - | - | 22 |
| 0.1%S3  AgNPc | 78 |  | 0,1 | 22.9 |
| 1%D1 3mL  AgNPsol | 78 | 1 |  | 22 |
| 1%D1 5mL  AgNPc | 78 | 1 | - | 22 |

**Supplementary Table 2.** The behavior of treated and untreated beech wood following immersion in water

| Time | L | L-MPTMS-Ag100 | Base coat  acrylic resin | 0.1%S3  AgNPc | 1%D1 3mL  AgNPsol | 1%D1 5mL  AgNPc |
| --- | --- | --- | --- | --- | --- | --- |
| 1 hour | 9.37 | 17.65 | 8.11 | 2.63 | 5.71 | 6.67 |
| 2 hours | 12.50 | 23.53 | 8.11 | 2.63 | 2.86 | 6.67 |
| 24 hours | 25.00 | 47.06 | 10.81 | 7.89 | 8.57 | 13.33 |
| 48 hours | 53.12 | 94.12 | 24.32 | 13.16 | 17.14 | 30.00 |
| 72 hours | 71.87 | 129.41 | 32.43 | 23.68 | 25.71 | 40.00 |
| 96 hours | 100.00 | 164.71 | 54.05 | 39.47 | 31.43 | 53.33 |

**Supplementary Table 3.** Evaluation of the antimicrobial activity of the polyacrylic resin solutions by the adapted disk diffusion method.

|  | Inhibition zone diameter (mm) | Arbitrary unit |
| --- | --- | --- |
| NS5R | | |
| 1% D1 3ml AgNPsol | 18 mm | 2 |
| 1% D1 5ml AgNPc | 17 mm | 2 |
| 0.1% S3 AgNPc | 15 mm | 2 |
| Base coat acrylic resin | 14 mm | 2 |
| C161516 | | |
| 1% D1 3ml AgNPsol | 11 mm | 2 |
| 1% D1 5ml AgNPc | 13 mm | 2 |
| 0.1% S3 AgNPc | 10 mm | 1 |
| Base coat acrylic resin | 11 mm | 2 |

**Supplementary Table 4.** The MIC and the corresponding subinhibitory concentrations values (MIC/2 and MIC/4) of the tested polyacrylic resin solutions on the biodeteriogenic microbial strains.

|  | |  | MIC (%) | |  |  |
| --- | --- | --- | --- | --- | --- | --- |
|  | Mean | Std. dev. | MIC/2 (%) | MIC/4 (%) |  |  |
| bacterial strains | **NS5-R** |  |  |  |  |  |
|  | Base coat acrylic resin | 2.04 | 0.83 | 1.02 | 0.51 |  |
|  | 0.1% S3 AgNPc | 2.52 | 0.83 | 1.26 | 0.63 |  |
|  | 1% D1 3 mL AgNPsol | 1.56 | 0 | 0.78 | 0.39 |  |
|  | 1% D1 5 mL AgNPc | 2.04 | 0.83 | 1.02 | 0.51 |  |
|  | **C16156** |  |  |  |  |  |
|  | Base coat acrylic resin | 2.52 | 0.83 | 1.26 | 0.63 |  |
|  | 0.1% S3 AgNPc | 4.08 | 1.88 | 2.04 | 1.02 |  |
|  | 1% D1 3 mL AgNPsol | 2.52 | 0.83 | 1.26 | 0.63 |  |
|  | 1% D1 5 mL AgNPc | 1.56 | 0 | 0.78 | 0.39 |  |
| fungal strains | **NS4-2B** |  |  |  |  |  |
|  | Base coat acrylic resin | 20.83 | 3.61 | 10.42 | 5.21 |  |
|  | 0.1% S3 AgNPc | 20.83 | 3.61 | 10.42 | 5.21 |  |
|  | 1% D1 3 mL AgNPsol | 20.83 | 3.61 | 10.42 | 5.21 |  |
|  | 1% D1 5 mL AgNPc | 25 | 0 | 12.50 | 6.25 |  |
|  | **NS11C** |  |  |  |  |  |
|  | Base coat acrylic resin | 12.5 | 0 | 6.25 | 3.13 |  |
|  | 0.1% S3 AgNPc | 6.25 | 0 | 3.13 | 1.56 |  |
|  | 1% D1 3 mL AgNPsol | 8.33 | 1.80 | 4.17 | 2.08 |  |
|  | 1% D1 5 mL AgNPc | 2.34 | 0.68 | 1.17 | 0.59 |  |

**Supplementary Table 5.** The PICA% values for the corresponding subinhibitory concentrations (MIC/2 and MIC/4) of the tested polyacrylic resin solutions on the biodeteriogenic microbial strains.

| PICA% | | | | | | |  |
| --- | --- | --- | --- | --- | --- | --- | --- |
|  | MIC/2 | | | MIC/4 | | |  |
|  |  | Mean | Std. dev. | p-value | Mean | Std. dev. | p-value |
| bacterial strains | **NS5-R** |  |  |  |  |  |  |
|  | Strain control | **100** | **13.31** |  | **100** | **13.31** |  |
|  | Base coat acrylic resin | 7.15 | 2.54 | <0,0001 | 43.13 | 7.27 | 0.0055 |
|  | 0.1% S3 AgNPc | 14.32 | 18.40 | <0,0001 | 14.07 | 7.64 | <0,0001 |
|  | 1% D1 5 mL AgNPc | 11.96 | 9.54 | <0,0001 | 20.79 | 12.44 | <0,0001 |
|  | **C16156** |  |  |  |  |  |  |
|  | Strain control | **100** | **16.24** |  | **100** | **16.24** |  |
|  | Base coat acrylic resin | 58.29 | 6.36 | 0.0659 | 7.50 | 2.54 | <0,0001 |
|  | 0.1% S3 AgNPc | 111.50 | 7.38 | 0.9582 | 112.26 | 10.05 | 0.9442 |
|  | 1% D1 5 mL AgNPc | 11.92 | 5.26 | <0,0001 | 13.92 | 0.85 | <0,0001 |
| fungal strains | **NS4-2B** |  |  |  |  |  |  |
|  | Strain control | **100** | **13.47** |  | **100** | **13.47** |  |
|  | Base coat acrylic resin | 247.64 | 39.38 | <0,0001 | 288.32 | 39.38 | <0,0001 |
|  | 0.1% S3 AgNPc | 244.27 | 17.72 | <0,0001 | 276.27 | 41.23 | <0,0001 |
|  | 1% D1 5 mL AgNPc | 221.72 | 28.22 | <0,0001 | 277.15 | 28.22 | <0,0001 |
|  | **NS11C** |  |  |  |  |  |  |
|  | Strain control | **100** | **15.04** |  | **100** | **15.04** |  |
|  | Base coat acrylic resin | 206.25 | 28.05 | <0,0001 | 248.48 | 41.80 | <0,0001 |
|  | 0.1% S3 AgNPc | 128.86 | 13.73 | 0.1843 | 244.98 | 38.98 | <0,0001 |
|  | 1% D1 5 mL AgNPc | 18.36 | 5.58 | <0,0001 | 10.65 | 2.05 | <0,0001 |

**Supplementary Table 6.** The influence of the polyacrylic resin solutions at the corresponding subinhibitory MIC/2 and MIC/4 concentrations on the enzymatic and organic acids production in the selected bacterial strains.

| MIC/2 | | | | | | | | | | | | |
| --- | --- | --- | --- | --- | --- | --- | --- | --- | --- | --- | --- | --- |
|  | **Esterase TW20** | | | **Esterase TW80** | | | **Organic acids** | | | **Amylase** | | |
|  | **Mean** | **Std. dev.** | **p-value** | **Mean** | **Std. dev.** | **p-value** | **Mean** | **Std. dev.** | **p-value** | **Mean** | **Std. dev.** | **p-value** |
| NS 5 R | **100** | **12.5** |  | **100** | **8.66** |  | **100** | **12.37** |  | **100** | **24.74** |  |
| Base coat acrylic resin | 79.17 | 7.22 | 0.3855 | 115 | 17.32 | 0.9745 | 57.14 | 24.74 | 0.0156 | 71.43 | 24.74 | 0.2955 |
| 0.1 S3 AgNPc | 188.89 | 19.25 | <0,0001 | 95 | 8.66 | 0.9997 | 142.86 | 12.37 | 0.0156 | 114.29 | 24.74 | 0.9095 |
| 1% D1 3mL AgNPsol | 66.67 | 0 | 0.0432 | 95 | 8.66 | <0,0001 | 57.14 | 32.73 | 0.0156 | 123.08 | 13.32 | 0.5238 |
| 1% D1 5mL AgNPc | 91.67 | 28.87 | 0.9827 | 150 | 15 | 0.0911 | 92.86 | 12.37 | 0.9968 | 114.29 | 24.74 | 0.9095 |
| C 16156B | **100** | **0** |  | **100** | **10.19** |  | **100** | **10.19** |  | **100** | **13.32** |  |
| Base coat acrylic resin | 122.22 | 19.25 | 0.3179 | 111.76 | 10.19 | 0.9933 | 100 | 20.38 | >0,9999 | 100 | 13.32 | 0.9095 |
| 0.1 S3 AgNPc | 79.17 | 7.22 | 0.3855 | 123.53 | 17.65 | 0.7885 | 111.76 | 20.38 | 0.9412 | 115.38 | 0 | >0,9999 |
| 1% D1 3mL AgNPsol | 104.17 | 7.22 | 0.9995 | 0.00 | 0.00 | 0.9997 | 100 | 10.19 | >0,9999 | 114.29 | 24.74 | 0.2259 |
| 1% D1 5mL AgNPc | 100 | 0 | >0,9999 | 123.53 | 0 | 0.7885 | 100 | 10.19 | >0,9999 | 100 | 13.32 | 0.2955 |
| MIC/4 | | | | | | | | | | | | |
|  | **Esterase TW20** | | | **Esterase TW80** | | | **Organic acids** | | | **Amylase** | | |
|  | **Mean** | **Std. dev.** | **p-value** | **Mean** | **Std. dev.** | **p-value** | **Mean** | **Std. dev.** | **p-value** | **Mean** | **Std. dev.** | **p-value** |
| NS 5 R | **100** | **12.5** |  | **100** | **8.66** |  | **100** | **12.37** |  | **100** | **24.74** |  |
| Base coat acrylic resin | 87.5 | 0 | 0.8624 | 110 | 17.32 | 0.9971 | 50 | 32.73 | 0.0032 | 85.71 | 42.86 | >0,9999 |
| 0.1 S3 AgNPc | 188.89 | 19.25 | <0,0001 | 105 | 15 | 0.9997 | 150 | 21.43 | 0.0032 | 100 | 24.74 | 0.8741 |
| 1% D1 3mL AgNPsol | 88.89 | 19.25 | 0.9186 | 115 | 8.66 | <0,0001 | 85.71 | 21.43 | 0.8567 | 130.77 | 13.32 | 0.9095 |
| 1% D1 5mL AgNPc | 83.33 | 7.22 | 0.6269 | 135 | 15 | 0.3897 | 92.86 | 24.74 | 0.9968 | 71.43 | 24.74 | >0,9999 |
| C 16156B | **100** | **0** |  | **100** | **10.19** |  | **100** | **10.19** |  | **100** | **13.32** |  |
| Base coat acrylic resin | 111.11 | 19.25 | 0.9186 | 105.88 | 0 | 0.9996 | 82.35 | 10.19 | 0.6926 | 115.38 | 0 | 0.8741 |
| 0.1 S3 AgNPc | 75.00 | 12.50 | 0.2077 | 129.41 | 10.19 | 0.5793 | 100 | 26.96 | >0,9999 | 107.69 | 13.32 | 0.9969 |
| 1% D1 3mL AgNPsol | 104.17 | 7.22 | 0.9995 | 0.00 | 0.00 | 0.9745 | 88.24 | 0 | 0.9412 | 85.71 | 0 | 0.9095 |
| 1% D1 5mL AgNPc | 100 | 0 | >0,9999 | 111.76 | 10.19 | 0.9933 | 82.35 | 20.38 | 0.6926 | 115.38 | 0 | 0.8741 |

**Supplementary Table 7.** The influence of the polyacrylic resin solutions at the corresponding subinhibitory MIC/2 and MIC/4 concentrations on the enzymatic and organic acids production in the selected fungal strains.

| MIC/2 | | | | | | | | | | | | | | | |
| --- | --- | --- | --- | --- | --- | --- | --- | --- | --- | --- | --- | --- | --- | --- | --- |
|  | **Caseinase** | | | **Esterase TW20** | | | **Esterase TW80** | | | **Organic acids** | | | **Amylase** | | |
|  | **Mean** | **Std. dev.** | **p-value** | **Mean** | **Std. dev.** | **p-value** | **Mean** | **Std. dev.** | **p-value** | **Mean** | **Std. dev.** | **p-value** | **Mean** | **Std. dev.** | **p-value** |
| NS4-2B | **100** | **12.37** |  | **100** | **24.74** |  | **100** | **5.41** |  | **100** | **30.05** |  | **100** | **15.75** |  |
| Base coat acrylic resin | 82.14 | 26.96 | 0.4368 | 78.57 | 12.37 | 0.3535 | 43.75 | 5.41 | 0.043 | 116.22 | 18.72 | 0.7673 | 118.18 | 15.75 | 0.7596 |
| 0.1 S3 AgNPc | 28.57 | 16.37 | <0,0001 | 0 | 0 | <0,0001 | 0 | 0 | <0,0001 | 63.89 | 20.97 | 0.0582 | 163.64 | 0 | 0.0006 |
| 1% D1 3mL AgNPsol | 103.57 | 6.19 | 0.9995 | 78.57 | 12.37 | 0.3555 | 31.25 | 5.41 | 0.0077 | 97.22 | 4.81 | 0.9997 | 0 | 0 | <0,0001 |
| 1% D1 5mL AgNPc | 75 | 18.56 | 0.1345 | 92.86 | 12.37 | 0.9928 | 53.13 | 5.41 | 0.1286 | 102.78 | 4.81 | 0.9997 | 118.18 | 15.75 | 0.7596 |
| NS11C | **100** | **6.93** |  | **100** | **20** |  | **100** | **18.23** |  | **100** | **4.68** |  | **100** | **0** |  |
| Base coat acrylic resin | 80 | 18.33 | 0.3194 | 86.67 | 11.55 | 0.8216 | 84.21 | 9.12 | 0.9661 | 136.11 | 4.81 | 0.0582 | 108.33 | 28.87 | 0.9944 |
| 0.1 S3 AgNPc | 28 | 6.93 | <0,0001 | 80 | 0 | 0.4297 | 42.11 | 9.12 | 0.0349 | 81.08 | 8.11 | 0.6238 | 33.33 | 14.43 | 0.0003 |
| 1% D1 3mL AgNPsol | 75 | 18.56 | >0,9999 | 46.67 | 11.55 | 0.0003 | 42.11 | 9.12 | 0.0349 | 89.19 | 8.11 | 0.9622 | 75 | 25 | 0.4358 |
| 1% D1 5mL AgNPc | 120 | 0 | 0.3194 | 66.67 | 11.55 | 0.0432 | 73.68 | 9.12 | 0.6921 | 83.78 | 4.68 | 0.7673 | 58.33 | 14.43 | 0.045 |
| MIC/4 | | | | | | | | | | | | | | | |
|  | **Caseinase** | | | **Esterase TW20** | | | **Esterase TW80** | | | **Organic acids** | | | **Amylase** | | |
|  | **Mean** | **Std. dev.** | **p-value** | **Mean** | **Std. dev.** | **p-value** | **Mean** | **Std. dev.** | **p-value** | **Mean** | **Std. dev.** | **p-value** | **Mean** | **Std. dev.** | **p-value** |
| NS4-2B | **100** | **12.37** |  | **100** | **24.74** |  | **100** | **5.41** |  | **100** | **30.05** |  | **100** | **15.75** |  |
| Base coat acrylic resin | 28.571 | 6.186 | <0,0001 | 114.29 | 12.37 | 0.7697 | 90.63 | 5.41 | 0.9974 | 110.81 | 4.68 | 0.9622 | 109.09 | 27.27 | 0.9798 |
| 0.1 S3 AgNPc | 78.571 | 6.186 | 0.254 | 171.43 | 21.43 | <0,0001 | 78.13 | 10.83 | 0.84 | 127.78 | 17.35 | 0.2236 | 145.45 | 15.75 | 0.0223 |
| 1% D1 3mL AgNPsol | 100 | 13.86 | 0.1345 | 78.571 | 12.37 | 0.3555 | 84.38 | 9.38 | 0.968 | 111.1 | 4.81 | 0.9563 | 0 | 0 | <0,0001 |
| 1% D1 5mL AgNPc | 28.571 | 6.186 | <0,0001 | 78.57 | 12.37 | 0.3535 | 115.63 | 10.83 | 0.968 | 111.11 | 4.81 | 0.9563 | 118.18 | 15.75 | 0.6962 |
| NS11C | **100** | **6.928** |  | **100** | **20** |  | **100** | **18.23** |  | **100** | **4.68** |  | **100** | **0** |  |
| Base coat acrylic resin | 116 | 6.928 | 0.5539 | 133.33 | 23.09 | 0.0432 | 168.42 | 50.76 | 0.0081 | 127.78 | 4.81 | 0.2236 | 75 | 0 | 0.4358 |
| 0.1 S3 AgNPc | 116 | 6.928 | 0.5539 | 133.33 | 11.55 | 0.0432 | 210.53 | 119.56 | <0,0001 | 110.81 | 20.40 | 0.9622 | 41.67 | 14.43 | 0.0017 |
| 1% D1 3mL AgNPsol | 112 | 13.86 | 0.8138 | 133.33 | 11.55 | 0.0432 | 142.11 | 41.78 | 0.2088 | 116.2 | 4.68 | 0.7673 | 83.33 | 28.87 | 0.8255 |
| 1% D1 5mL AgNPc | 116 | 13.86 | 0.5539 | 153.33 | 30.55 | 0.0003 | 200 | 9.12 | <0,0001 | 105.41 | 8.11 | 0.9994 | 58.33 | 14.43 | 0.045 |

**Supplementary Table 8.** Results of the microbial viability of the tested biodeteriogenic microbial strains in presence of wooden material treated with L-MPTMS and L-MPTMS_Ag100 and the corresponding PICA% values to the material and to the inert substratum.

|  |  | Microbial viability (%) | | | PICA% to the material | | | PICA% to the inert subtratum | | |
| --- | --- | --- | --- | --- | --- | --- | --- | --- | --- | --- |
|  | | Mean | Std. dev. | p-value | Mean | Std. dev. | p-value | Mean | Std. dev. | p-value |
| bacterial strains | Growth control **NS5-R** | **100** | **9.87** |  |  |  |  | **100** | **16.43** |  |
|  | Untreated material | 80.22 | 1.30 | 0.0148 | **100** | **1.22** |  | 154.69 | 21.78 | 0.0261 |
|  | L-MPTMS | 78.78 | 1.20 | 0.0077 | 109.38 | 1.69 | 0.0022 | 108.26 | 48.31 | 0.9689 |
|  | L-MPTMS_Ag100 | 30.66 | 1.58 | <0,0001 | 65.72 | 2.16 | <0,0001 | 77.62 | 5.64 | 0.6171 |
|  | Growth control **C16156** | **100** | **1.97** |  |  |  |  | **100** | **4.69** |  |
|  | Untreated material | 87.88 | 31.20 | 0.2833 | **100** | **7.66** |  | 130.98 | 27.58 | 0.344 |
|  | L-MPTMS | 76.06 | 2.85 | 0.0154 | 61.78 | 2.54 | <0,0001 | 122.32 | 28.79 | 0.6191 |
|  | L-MPTMS_Ag100 | 36.93 | 0.75 | <0,0001 | 56.92 | 1.61 | <0,0001 | 109.86 | 23.00 | 0.9489 |
| fungal strains | Growth control **NS4-2b** | **100** | **0.96** |  |  |  |  | **100** | **9.28** |  |
|  | Untreated material | 131.60 | 11.08 | 0.0149 | **100** | **1.90** |  | 143.61 | 28.75 | 0.1008 |
|  | L-MPTMS | 91.08 | 9.89 | 0.7568 | 75.24 | 1.58 | <0,0001 | 83.60 | 9.85 | 0.8069 |
|  | L-MPTMS_Ag100 | 111.35 | 13.45 | 0.6056 | 38.43 | 0.64 | <0,0001 | 94.51 | 8.25 | 0.9904 |
|  | Growth control **NS11C** | **100** | **2.69** |  |  |  |  | **100** | **5.73** |  |
|  | Untreated material | 119.78 | 0.12 | 0.1849 | **100** | **5.11** |  | 144.94 | 22.77 | 0.0868 |
|  | L-MPTMS | 110.66 | 4.54 | 0.6494 | 88.53 | 2.27 | 0.0003 | 83.66 | 20.99 | 0.8086 |
|  | L-MPTMS_Ag100 | 120.54 | 6.02 | 0.1618 | 68.66 | 1.45 | <0,0001 | 123.92 | 26.78 | 0.5652 |

**Supplementary Table 9.** Results of the microbial viability of the tested biodeteriogenic microbial strains in presence of wooden material treated with synthetic resins and the corresponding PICA% values to the material and to the inert substratum.

|  |  | Microbial viability(%) | | | PICA% to the material | | | PICA% to the inert subtratum | | |  |
| --- | --- | --- | --- | --- | --- | --- | --- | --- | --- | --- | --- |
|  |  | Mean | Std. dev. | p-value | Mean | Std. dev. | p-value | Mean | Std. dev. | p-value |  |
|  | **BACTERIAL STRAINS** | Growth control **NS5-R** | **100** | **0.87** |  |  |  |  | **100** | **16.43** |  |
|  |  | Untreated material | 80.22 | 1.30 | 0.07 | **100** | **1.22** |  | 154.69 | 21.78 | 0.0019 |
|  |  | Base coat acrylic resin | 93.82 | 3.93 | 0.9527 | 50.13 | 1.42 | <0,0001 | 103.70 | 17.50 | 0.9998 |
|  |  | 0.1% S3 AgNPc | 107.89 | 1.08 | 0.8763 | 125.97 | 6.11 | <0,0001 | 97.86 | 12.68 | >0,9999 |
|  |  | 1% D1 3 mL AgNPsol | 98.05 | 3.94 | 0.9998 | 103.88 | 3.29 | 0.4725 | 97.66 | 5.05 | >0,9999 |
|  |  | 1% D1 5 mL AgNPc | 98.75 | 1.65 | >0,9999 | 114.58 | 3.43 | <0,0001 | 91.40 | 1.88 | 0.9865 |
|  |  | Growth control **C16156** | **100** | **3.26** |  |  |  |  | **100** | **4.69** |  |
|  |  | Untreated material | 87.88 | 31.20 | 0.37 | **100** | **7.66** |  | 130.98 | 27.58 | 0.1991 |
|  |  | Base coat acrylic resin | 91.53 | 4.45 | 0.8396 | 65.99 | 1.45 | <0,0001 | 141.98 | 23.71 | 0.0301 |
|  |  | 0.1% S3 AgNPc | 108.77 | 3.01 | 0.8194 | 91.26 | 2.99 | 0.0147 | 141.15 | 11.90 | 0.0353 |
|  |  | 1% D1 3 mL AgNPsol | 86.19 | 0 | 0.3835 | 96.18 | 2.59 | 0.4872 | 121.88 | 23.47 | 0.5695 |
|  |  | 1% D1 5 mL AgNPc | 88.43 | 3.59 | 0.5823 | 87.83 | 0.16 | 0.0005 | 116.68 | 1.92 | 0.8053 |
|  | **FUNGAL STRAINS** | Growth control **NS4-2b** | **100** | **4.51** |  |  |  |  | **100** | **9.28** |  |
|  |  | Untreated material | 131.60 | 11.08 | 0.03 | **100** | **1.90** |  | 143.61 | 28.75 | 0.0217 |
|  |  | Base coat acrylic resin | 110.27 | 9.10 | 0.9096 | 80.88 | 2.55 | <0,0001 | 111.91 | 10.96 | 0.9448 |
|  |  | 0.1% S3 AgNPc | 106.06 | 18.55 | 0.9906 | 76.21 | 2.62 | <0,0001 | 98.57 | 19.25 | >0,9999 |
|  |  | 1% D1 3 mL AgNPsol | 108.65 | 10.42 | 0.9548 | 83.12 | 3.70 | <0,0001 | 144.78 | 6.83 | 0.0171 |
|  |  | 1% D1 5 mL AgNPc | 98.15 | 15.18 | >0,9999 | 66.90 | 2.40 | <0,0001 | 111.51 | 1.89 | 0.9521 |
|  |  | Growth control **NS11C** | **100** | **1.52** |  |  |  |  | **100** | **5.73** |  |
|  |  | Untreated material | 119.78 | 0.12 | 0.37 | **100** | **5.11** |  | 144.94 | 22.77 | 0.0165 |
|  |  | Base coat acrylic resin | 87.41 | 10.34 | 0.8096 | 86.82 | 3.43 | 0.0002 | 129.53 | 9.71 | 0.2439 |
|  |  | 0.1% S3 AgNPc | 94.66 | 10.48 | 0.9948 | 89.25 | 5.50 | 0.0022 | 153.68 | 10.23 | 0.0024 |
|  |  | 1% D1 3 mL AgNPsol | 97.99 | 9.61 | >0,9999 | 84.42 | 1.29 | <0,0001 | 130.31 | 19.39 | 0.2188 |
|  |  | 1% D1 5 mL AgNPc | 98.84 | 4.65 | >0,9999 | 87.39 | 1.41 | 0.0003 | 121.23 | 21.36 | 0.6007 |


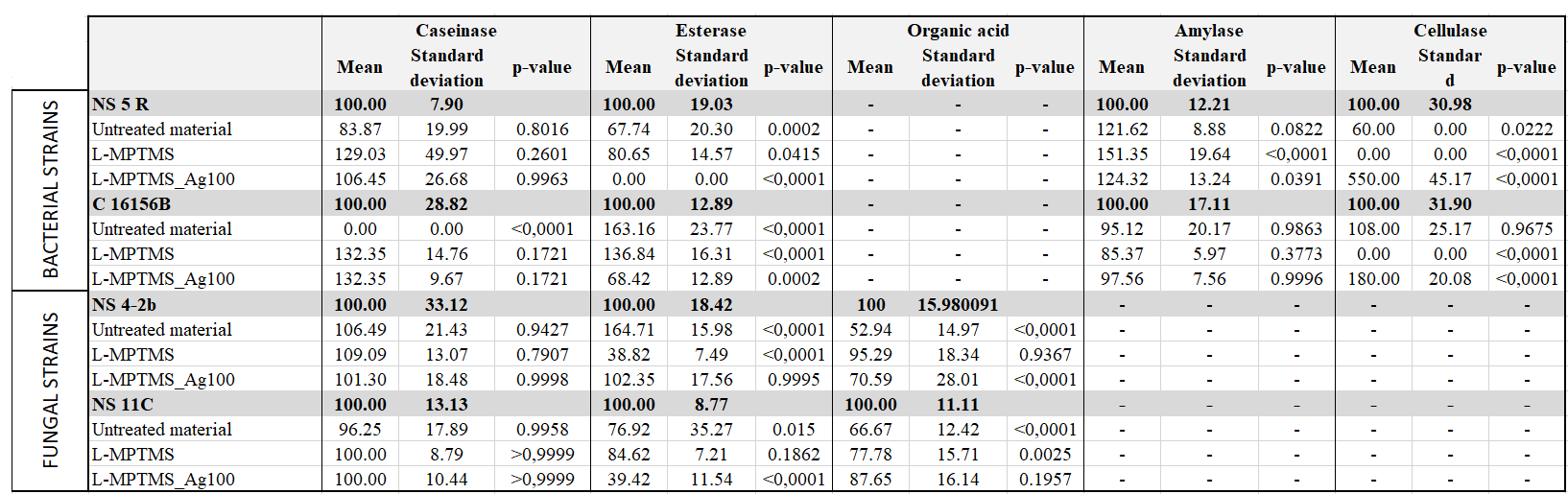
**Supplementary Table 10.** The influence of wooden material treated with L-MPTMS and L-MPTMS-Ag100 on the enzymatic and organic acids production in selected bacterial and fungal strains.


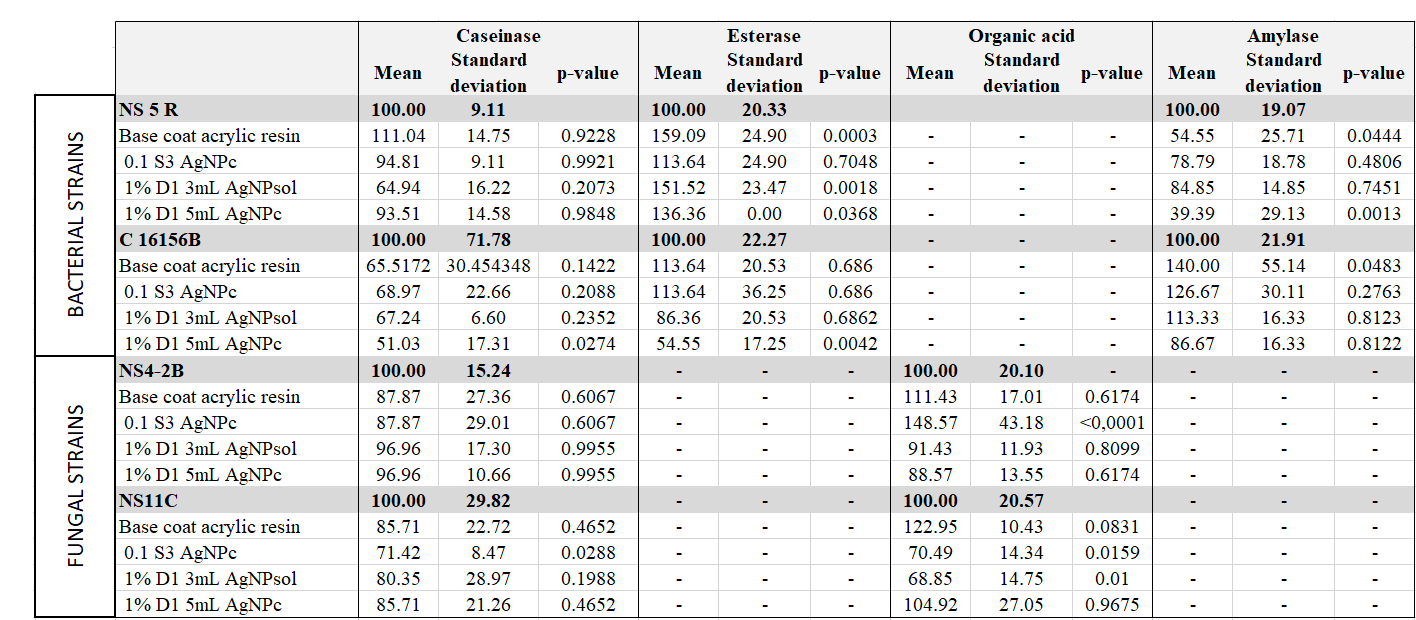
**Supplementary Table 11.** The influence of wooden material treated with synthetic resins on the enzymatic and organic acids production in selected bacterial and fungal strains.
